# Changes in air composition driven by differences in biofuel consumption

**DOI:** 10.1007/s11356-026-37733-9

**Published:** 2026-04-20

**Authors:** Camila Novais Farias, Débora Pagliuso, Adriana Grandis, José Vinícius Martins, Clara Rodrigues Pereira, Lilian Lefol Nani Guarieiro, Paulo Eduardo Artaxo-Netto, Marcos Silveira Buckeridge, Pérola de Castro Vasconcellos

**Affiliations:** 1 Department of Chemistry, Institute of Chemistry, University of São Paulo, São Paulo, 05508-000 Brazil; 2 Department of Botany, Institute of Biosciences, University of São Paulo, São Paulo, 05508-090 Brazil; 3 Department of Mineralogy and Geotectonics, Institute of Geosciences, University of São Paulo, São Paulo, 05508-080 Brazil; 4 SENAI CIMATEC University, Salvador, Bahia 41650-010 Brazil; 5 Department of Applied Physics, Institute of Physics, University of São Paulo, São Paulo, 05508-090 Brazil

**Keywords:** PM_2.5_, Air pollution, PAHs, Metals, Source apportionment

## Abstract

In São Paulo, the largest city in the Southern Hemisphere, the air pollution is mainly associated with vehicular traffic. The use of biofuels gives unique characteristics to the Brazilian vehicle fleet. In this study, PM_2.5_ samples were collected in the city at the beginning of the lockdown (from March to August 2020) and in the same period in 2022 (*n* = 93 samples), after the full economic reopening. The source apportionment by the FA-MLR approach showed that vehicular traffic, biomass burning, and secondary formation contributed to PM_2.5_ concentrations. Biomass burning made remarkable contributions in both years, and was the main source in 2020. Vehicular traffic was the primary source after the economic reopening. In 2020, concentrations of most pollutants (water-soluble ions, elements, PAHs, and their derivatives) decreased compared to previous studies, but the air pollution in 2022 reached levels close to the pre-pandemic. Additionally, Cu and Mo, elements linked to vehicular traffic, unexpectedly decreased in 2022 in relation to the lockdown and pre-pandemic period. These results were associated with the decrease in ethanol (and consequently, increase in gasoline) consumption in flexible-fuel vehicles, due to increased fuel prices. Biomass burning markers (Rb, retene, and anhydride monosaccharides) showed no change during and after the pandemic, indicating that this activity was not affected by social distancing measures. The resurgence of anthropogenic activities in 2022 resulted in increasing health risks due to exposure to carcinogenic species.

**Supplementary information:**

The online version contains supplementary material available at 10.1007/s11356-026-37733-9.

## Introduction

São Paulo is the largest city in Brazil and in the Southern Hemisphere (over twelve million inhabitants) (IBGE 2022), and often experiences high levels of air pollution. Studies have shown that several sources contribute to pollutant emissions in this metropolis, including vehicular traffic, biomass burning, and industrial activities (Pereira et al. 2025; Andrade et al. 2017). Traffic emissions are often the main source of pollutants in São Paulo city, along with those from biomass burning that release specific markers (retene and anhydrous monosaccharides) (Serafeim et al. 2023; Pereira et al. 2025).

The program for the control of air pollution from motor vehicles was implemented by the National Environmental Council (CONAMA 1986) to define limits for pollutant emissions and to promote their gradual reduction. In 2022, recommendations (new targets) came into effect for light and heavy vehicles, establishing the reduction of nitrogen oxides and fine particulate matter emissions.

The Brazilian vehicle fleet relies heavily on biofuels, using blends of gasoline and anhydrous ethanol (gasohol), as well as diesel and biodiesel. Hydrated ethanol is used as pure fuel in ethanol-powered or flex-fuel vehicles (ANP 2023). It imparts unique emissions profiles to this fleet. Previous studies have shown that the use of biofuels is associated with lower PAH emission factors compared to the combustion of other fuels (Abrantes et al. 2009; Pereira et al. 2023). Copper was found to be a marker for ethanol/gasohol combustion (Sánchez-Ccoyllo et al. 2009; Pereira et al. 2023). In addition, the use of ethanol or gasohol by flex-fuel vehicles is influenced by the fuel prices.

In 2022, there was the complete reopening of the economy after the COVID-19 pandemic, with the return of in-person activities. The COVID-19 pandemic caused changes in human activity and global air pollution in 2020 (WHO 2020; Zhu et al. 2020). In São Paulo, social restriction measures were implemented in March 2020 and referred to as a lockdown (GESP 2020). From March to April 2020, Nakada and Urban (2020) reported a rise in ozone levels and a decrease in other pollutants (SO_2_, NO, NO_x_, NO_2_, CO, PM_10_, and PM_2.5_) in the city center and at two road sites in São Paulo. However, the easing of social restriction measures at the end of 2020 caused NO_x_ levels to rebound to values near pre-pandemic levels (Pérez-Martínez et al. 2022).

Particles with diameters ≤2.5 µm (PM_2.5_) represent a significant concern for human health due to their penetration capability in pulmonary alveoli (Thangavel et al. 2022). Fine and ultrafine particles, along with their components, are associated with adverse health effects, such as loss of lung function in patients with asthma, oxidative stress, and formation of DNA adducts that lead to the development of cancer (Wyzga and Rohr 2015; Alves et al. 2020). Several studies indicate a link between rising PM_2.5_ levels and an increase in COVID-19 cases and deaths (Coker et al. 2020; Zhang et al. 2020; Zhou et al. 2021; Ibarra-Espinosa et al. 2022).

Several approaches are used to identify pollutant sources, such as Unmix, chemical mass balance (CMB), positive matrix factorization (PMF), principal component analysis (PCA), and factor analysis (FA). Factor analysis is a statistical method that separates the factors that explain the variability of the data (potential sources), and, similar to PCA, this approach is combined with an unnormalized solution (absolute principal component scores, APCS) and multiple linear regression (FA-MLR) to provide the quantitative assessment of the source contributions to PM (Hopke et al. 2020; Rahman and Thurston 2022). The minimum residual (MINRES) factor extraction method is a multivariate statistical method that provides robust results even with a small number of samples (Grooten et al. 2019).

In this study, the post-pandemic air pollution was compared with the lockdown period and pre-pandemic studies. The COVID-19 pandemic was an uncommon event that allowed the assessment of the impacts of social restriction measures on the air quality of a megacity that often displays air pollution-related issues. PM_2.5_ samples were collected in São Paulo immediately after the lockdown decree, for five months (autumn and winter) in 2020 and in the same period in 2022 (after full economic reopening), aiming to evaluate changes in composition, source patterns, and health exposure. The factor analysis and multiple linear regression (FA-MLR) approach was applied to identify the sources of PM_2.5_.

## Methodology

### PM_2.5_ sampling and meteorological data

Sample collection was carried out at the University of São Paulo (Fig. S1). The sampling took place during the cold season (autumn to winter). Climatological data (precipitation, relative humidity, temperature, direction, and wind speed) were obtained from the University Meteorological Station (IAG 2022) and are summarized in Table S1.

Samples of fine particulate matter (PM_2.5_) were collected from March 26th to August 31 st in 2020 (pandemic lockdown) and in 2022 (after complete economic reopening). A high-volume sampler (Energética, Brazil) at a flow of 1.13 m^3^ min^−1^ and quartz fiber filters (Whatman, USA) were used. Before sampling, the filters were decontaminated by heating in a muffle furnace at 550 °C for 5 h, then weighed in the ambient with controlled humidity (50%). Ninety-three samples (24 h-sampling, every 3 days) were obtained, along with 9 blank filters. The collected samples were weighed, wrapped in aluminum foil, packed in sealed polyethylene bags, and stored under refrigeration until analysis.

### Determination of organic and elemental carbon

The determination of carbonaceous species was performed with 1 cm^2^ punches from filters by thermo-optical analysis (Sunset Inc.) using the EUSAAR_2 method (Cavalli and Putaud 2010). The organic carbon (OC) was measured by desorbing different fractions of OC from the filters in an inert atmosphere (He) with a gradual heating process. Subsequently, elemental carbon (EC) was identified by heating under a gas flow containing 2% oxygen. The initial EC fraction included the pyrolyzed OC and EC. The detection limits were 0.2 µg C cm^−2^ for EC and 0.4 µg C cm^−2^ for OC, as specified by the manufacturer (Brown et al. 2019).

### Determination of water-soluble ions

Water-soluble ions (WSIs) were determined by ion chromatography with conductometric detection (Methrom AG, Switzerland). Punches (5 cm^2^) were extracted with 10 mL of milli-Q water in a polyethylene bottle, under ultrasonic agitation for 15 min (Pereira et al. 2025). The extracts were filtered with syringe filters (Millipore, PVDF, 0.45 µm), stored in polyethylene bottles, and frozen until analysis.

Sodium, lithium, ammonium, potassium, calcium, and magnesium cations were determined on a Metrosep C4 column (250 × 4.0 mm) with a buffer solution of dipicolinic acid (0.7 mmol L^−1^) and nitric acid (1.7 mmol L^−1^) as eluent. The inorganic anions chloride, fluoride, bromide, nitrate, sulfate, phosphate, nitrite, and the organic anions oxalate, succinate, and maleate were analyzed on a Metrosep A Supp 5 column (250 × 4.0 mm) with the buffer solution Na_2_CO_3_/NaHCO_3_ (3.2 and 1.0 mmol L^−1^, respectively) as the eluent.

External standards of cations and anions (Sigma-Aldrich, Switzerland), ranging from 0.1 to 3.6 µg mL^−1^, were prepared by diluting certified standards. Standard solutions containing Li^+^, Na^+^, K^+^, Mg^2+^, and Ca^2+^ (10 µg mL^−1^), ammonium (1000 µg mL^−1^), multi-anion solutions containing Cl^−^, SO_4_^2−^, F^−^, NO_3_^−^, PO_4_^3−^, and Br^−^ (10 µg mL^−1^), and individual standards for succinate, maleate, oxalate, and formate (1000 µg mL^−1^) were used. All samples were analyzed in duplicate. Limits of detection (DLs) ranged from 0.01 to 0.076 µg mL^−1^ for anions and 0.02 to 0.049 µg mL^−1^ for cations (Table S2).

### Determination of trace elements and enrichment factors calculations

Trace elements were extracted according to the methodology described by Pereira and collaborators (2023). Briefly, a 25 cm^2^ section from each sample and blank was extracted with 25 mL of a water and HNO_3_ (3:2) mixture in a microwave digestion system (CEM MDS-2000, USA). The determination was carried out using ICP-MS (model iCAP-Q, Thermo Fisher, USA). External standards with concentrations from 0 to 150 ng mL^−1^ were prepared by diluting stock solutions (Specsol, Brazil) with 10% HNO_3_. The metals determined included Fe, Mn, Zn, Ti, V, As, Pb, Mo, Cd, Sn, Sb, Rb, Cr, Cu, Se, Sr, Ba, and Ni. The DLs varied from 0.004 to 1.670 ng g^−1^ (Table S2).

Enrichment factors (EFs) of metals in PM_2.5_ were estimated using the method described by Gelado-Caballero et al. (2012). EFs were calculated to determine which elements presented higher abundance in PM_2.5_ in comparison to the Earth’s crust, indicating the influence of anthropogenic emissions. Fe was considered the reference element, due to its greater abundance in the crust. The EFs were calculated as described in Eq. 1:

1 $$EF=\frac{\frac{{[X]}_{PM2.5}}{{[Fe]}_{PM2.5}}}{\frac{{[X]}_{crust}}{{[Fe]}_{crust}}}$$

where EF is the enrichment factor; [X]_PM2.5_ is the concentration of the element “X” in PM_2.5_; [X]_crust_ is the abundance of the element “X” in the crust; [Fe]_PM2.5_ is the concentration of Fe in PM_2.5_; [Fe]_crust_ is the abundance of Fe in the crust. The relative abundances of elements in the Earth’s crust were obtained from Lee (1999). Natural sources were considered predominant for EF values lower than 10, while EF ≥ 10 indicates the predominance of anthropogenic emissions.

### Determination of monosaccharides

Anhydride monosaccharides (levoglucosan, mannosan, and galactosan) were measured following the method adapted from Gonçalves and collaborators (2021). Punches (8 cm^2^) of the filters were extracted with ultrapure water by ultrasonic agitation (30 min). To concentrate the samples, the aqueous extracts were frozen, lyophilized, and reconstituted in 500 µL of water. The extracts were centrifuged, and the supernatants were filtered through PVDF filters (Millipore, 0.22 µm) to identify levoglucosan, mannosan, and galactosan.

Primary monosaccharides (glucose, arabinose, xylose, rhamnose, fucose, galactose, and mannose) were determined following the methodology described by Pereira and collaborators (2021). To ensure the complete quantification of monosaccharides present in plant and microorganism cell walls, the decanted particles were hydrolyzed with 1 mL of 2 N TFA (trifluoroacetic acid) for 1 h at 100 °C. After hydrolysis, the samples were dried in a vacuum centrifuge, resuspended in 500 μL of ultrapure water, and filtered (PVDF, 0.22 µm). The analyses were conducted using anion-exchange chromatography with a pulsed amperometric detector (HPAEC-PAD), using a CarboPac SA10 column (ICS 5000 system, Dionex-Thermo) and isocratic elution with 99.2% H_2_O/0.8% NaOH (1 mL min^−1^). Detection of the analytes was carried out in a post-column containing 500 mmol L^−1^ NaOH (0.5 mL min^−1^), by amperometric detection. The detection limits varied from 0.3 to 0.6 µg mL^−1^ (Table S3).

### Determination of polycyclic aromatic hydrocarbons and their derivatives

The procedure for extracting and fractioning the samples for polycyclic aromatic hydrocarbons and derivatives (oxy and nitro-PAH) was adapted from Alves and collaborators (2017). Samples and blank filters (50 cm^2^) were spiked with deuterated chrysene and extracted by ultrasonic stirring with dichloromethane (3 × 60 mL, for 20 min). The extracts were fractionated in a chromatographic column filled with silica gel (2.5 g) eluted with 15 mL of a mixture of dichloromethane/acetone 2:1. The fraction containing the PAHs and the derivatives, oxy and nitro-PAHs, was concentrated and filtered with syringe filters (Chromastore, PTFE, 0.22 µm).

Analyses were conducted using gas chromatography coupled with mass spectrometry (Agilent, GC 7820 A, and MS 5975), with a DB-5 ms column (30 m × 0.250 mm, film thickness 0.25 µm) and helium gas (0.9997% purity) with a flow rate of 1.0 mL min^−1^. Injections were performed in splitless mode. The oven temperature program started at 80 °C, increasing at 10 °C min^−1^ to 100 °C. Then, it was heated at 8 °C min^−1^ up to 230 °C, followed by 2 °C min^−1^ up to 315 °C. The mass spectrometry detector was set to operate in electron impact mode (EI, 70 eV). The ion source temperature was kept at 250 °C, and the transfer line was set to 300 °C. Ion analysis was performed in SIM mode, monitoring two ions m/z per compound. Quantification was achieved using analytical curves generated with the EPA 610 standard mix (Sigma Aldrich, St. Louis, USA), containing the sixteen priority PAH of the US Environmental Protection Agency. The lighter PAHs—naphthalene (NAP), fluorene (FLU), acenaphthylene (ACY), acenaphthene (ACE), and anthracene (ANT)—were not quantified due to poor recovery (<75%). Standards of retene (RET), benzo(e)pyrene (BeP), coronene (COR), and perylene (PER), obtained from Sigma Aldrich (St. Louis, USA) and Fluka (St. Louis, USA), and oxy and nitro-PAH were also added. Standards for oxy-PAH 9,10-anthraquinone (9,10-ANQ), 9-fluorenone (9-FLO), 2-methylanthraquinone (2-MAQ), 7,12-benzo[a]anthraquinone (7,12-BaAQ), and 1,4-naphthoquinone (1,4-NPQ); and the nitro-HPA, 9-nitroanthracene (9-NANT), 1-nitropyrene (1-NPYR), 3-nitrofluoranthene (3-NFLT), and 6-nitrochrisene (6-NCHR) were purchased from Sigma Aldrich (St. Louis, USA). 2-nitrofluoranthene (2-NFLT) and 3-NFLT were determined together in the DB-5MS columns. Recoveries ranged from 87 to 130% for PAHs, 106 to 142% for oxy-PAHs, and 90 to 105% for nitro-PAHs. The limits of detection (DL) varied from 1.25 ng mL^−1^ to 10 ng mL^−1^, 5 to 20 ng mL^−1^, and 10 ng mL^−1^ to 15 ng mL^−1^ for the PAHs, oxy-PAHs, and nitro-PAHs, respectively (Table S4). All samples and external standards were analyzed in duplicate.

### Health risk assessment

The risk of environmental exposure to PAH for human health was assessed by the calculation of the benzo(a)pyrene-equivalent index (BaPE), which expresses the concentrations of carcinogenic PAH in terms of equivalent BaP concentration. This calculation involves multiplying the concentrations of carcinogenic PAHs by their respective carcinogenic potential relative to BaP, as described by Yassaa and collaborators (2001) (Eq. 2):

2 $$\mathrm{BaPE}=\left(\left[\mathrm{BaA}\right]\times0.06\right)+\left(\left[\mathrm{BbF}\right]\times0.07\right)+\left(\left[\mathrm{BkF}\right]\times0.07\right)+\left(\left[\mathrm{BaP}\right]\times1\right)+\left(\left[\mathrm{DBA}\right]\times0.6\right)+\left(\left[\mathrm{INP}\right]\times0.08\right)$$

The carcinogenic (BaP-TEQ) (Eq. 3) and mutagenic equivalents (BaP-MEQ) (Eq. 4) were estimated by multiplying the concentration of each PAH by its toxic equivalency factor (TEF) and mutagenic equivalency factor (MEF) relative to BaP (Nisbet and LaGoy 1992; Durant et al. 1996, 1999; Jung et al. 2010).

3 $$BaP-TEQ = ([BaA] \times 0.1) + ([CHR] \times 0.01)+([BbF] \times 0.1) + ([BkF] \times 0.1) + ([BaP] \times 1) + ([INP] \times 0.1) +([DBA] \times 5) + ([BPE] \times 0.01)$$

4 $$\begin{aligned}BaP-MEQ&=\left(\left[BaA\right]\times0.082\right)\\&+\left(\left[CHR\right]\times0.017\right)+\left(\left[BbF\right]\times0.25\right)\\&+\left(\left[BkF\right]\times0.11\right)+\left(\left[BaP\right]\times1\right)\\&+\left(\left[INP\right]\times0.31\right)+\left(\left[DBA\right]\times0.29\right)\\&+\left(\left[BPE\right]\times0.19\right)\end{aligned}$$

The incremental lifetime cancer risk from inhalation exposure to carcinogenic PAH (ILCR_inh_) was calculated using the method developed by the EPA (U.S. Epa 2009), as shown in Eq. 5:

5 $${\mathrm{ILCR}}_{\mathrm{inh}}=CSF\times\frac{BaP-TEQ\times IR\times EF\times ED\times ET}{BW\times AT}\times10^{-6}$$

where IR is the inhalation rate, 20 m^3^ day^−1^ for adults and 10 m^3^ day^−1^ for children (Soltani et al. 2015); EF is the frequency of exposure (365 days/year); ED is duration of exposure in years, 10 years for children and 70 years for adults; ET is the exposure time in (24 h/day); BW is the body mass of the exposed person, 15 kg for children and 70 kg for adults (Kamal et al. 2014); AT is the average time of exposure (70 years × 365 days/year × 24 h/day); and CSF is the inhalation cancer slope for BaP, 3.14 kg·day/mg (Wang et al. 2020).

To assess the health risks of metals inhalation in PM_2.5_, the exposure concentrations (EC_inh_) for children and adults were calculated according to Eq. 6 (Hu et al. 2012):

6 $${EC}_{inh}=\frac{C\times (ET\times EF\times ED)}{AT}$$

where C is the element concentration (µg m^−3^); exposure time (ET) is 24 h per day; frequency of exposure (EF) is 365 days per year; duration of the exposure (ED) is 6 years for children and 24 years for adults; and average exposure time (AT) is equal to ED × 365 days/year × 24 h/day for non-carcinogens and equal to 70 years × 365 days/year × 24 h/day for carcinogen risk.

The non-carcinogen risk (hazard quotient, HQ) and carcinogenic risk (CR) due to exposure to metals in PM_2.5_ were calculated by Eqs. 7 and 8:

7 $$HQ=\frac{{EC}_{inh}}{{R}_{f}C\times 1000}$$

8 $$CR={IUR\times EC}_{inh}$$

where RfC is the reference concentration (mg m^−3^) and IUR is the unit risk (µg m^−3^) recommended by U.S. EPA (2023) (Table S5).

### Validation and quality control

All glassware, tweezers, and cutters used were previously decontaminated with dichloromethane. The silica gel used in the sample’s fractionation was previously baked at 400 °C for 4 h. Analytical and grade reagents (purity ≥ 99%) were used. The blank filters were analyzed by the methodologies applied to the sample filters. The limits of detection and quantification for WSIs, monosaccharides, PAHs, and derivatives were determined by a visual method. For elements, DL and QL were calculated from parameters of the analytical curve (Shrivastava and Gupta 2011). Details are described in the supplementary information (SI).

Recovery rates were determined by enriching the filters with standards, followed by extraction using the same procedures applied to samples. The relative standard deviations of the measurements (RSD %) were determined as described in the SI (Tables S2–S4). All analytical curves were linear with R^2^ greater than 0.99.

### Statistical analysis

The Shapiro–Wilk test was conducted to assess the data normality. Since PM_2.5_ and most associated pollutants did not follow normal distribution (*p* > 0.05, Table S6), the correlations between variables were assessed using Spearman’s rank coefficients (Table S7 and S8), and the non-parametric Mann–Whitney test was applied to compare meteorological variables and pollutant concentrations during and after the pandemic (Table S6). The Spearman coefficients *(p* < *0.05*) were calculated using Statistica software (version 13.5.0.17, Tibco Software Inc). The Shapiro–Wilk and Mann–Whitney tests were performed in R Studio (Posit, version2023), using the *dplyr* and *rstatix* packages. The graphs were plotted using MS Excel (version 2019).

#### Backward air masses trajectories

The backward air masses’ trajectories were evaluated by the Hysplit (Hybrid Single-Particle Lagrangian Integrated Trajectory) model (Draxler and Rolph 2003) via the NOAA READY platform (National Oceanic and Atmospheric Administration). The meteorological data employed was the GDAS at 1-degree resolution. The trajectory running time was set to 72 h, with height levels set at 500, 1500, and 3000 m above ground level.

#### Source apportionment by Factor analysis-multiple linear regression

Factor analysis-multiple linear regression (FA-MLR) analysis was performed in R Studio, using the *psych* package. The default extraction method for the FA function in this package is the minimum residual (MINRES) (Revelle 2025). The MINRES is a robust factor extraction method that is not affected by deviations from normal distribution (Grooten et al. 2019). This model combines factor analysis (FA) with varimax rotation with the multiple linear regression (MLR) of PM_2.5_ concentrations on factor scores, to determine the sources’ contributions. The Bartlett’s test of sphericity and the Kaiser–Meyer–Olkin (KMO) test were applied to evaluate the suitability of the data for factor analysis. A KMO factor adequacy higher than 0.70 and *p-values* < 0.05 in the Bartlett’s test indicated the suitability of the data (Table S9).

The analysis was conducted separately for each sampling period, and 21 variables were included (Ti, V, Fe, Mn, Zn, Cu, As, Rb, Sr, Sb, As, Pb, NO_3_^−^, SO_3_^2−^, NH_4_^+^, nss-K^+^, Ca^2+^, oxalate, ∑PAHs, OC, and EC). The data were autoscaled before factor analysis (FA), and three factors were extracted using parallel analysis and the Scree plot (Fig. S2). Before the MLR analysis, the scores of FA were unnormalized following the procedure described by Thurston and Spengler (1985). The Cook’s distance method was used after regression to identify outliers and improve the model (Fig. S3). The contributions of the sources to PM_2.5_ concentrations were calculated as described by Hou and collaborators (2022) and detailed in the supplementary information.

## Results and discussion

### PM_2.5_

PM_2.5_ concentrations ranged from 5 to 35 µg m^−3^ (median = 17 µg m^−3^) from March to August in 2020, and from 6 to 71 µg m^−3^ (median = 24 µg m^−3^) during the same period in 2022 (Table 1). The Mann–Whitney test showed significantly higher concentrations in 2022 (*p* < *0.01*). PM_2.5_ levels during the lockdown period were also lower than those observed in the dry season (June to August) of the previous year campaign (2019), which was 22 µg m^−3^. Despite the reduction, the current limit recommended by the WHO (15 µg m^−3^, WHO 2021a) was exceeded in 27 days of sampling in 2020, whereas the 60 µg m^−3^ limit set by the National Environmental Council (CONAMA 2018) was not surpassed. After the economic reopening (2022), PM_2.5_ levels were comparable with those observed before the pandemic, and the concentrations exceeded the WHO guidelines on 36 days and the CONAMA on two sampling days.

**Table 1:** Concentrations of PM_2.5_, carbonaceous species (OC and EC), and water-soluble ion concentrations (WSI) in PM_2.5_ samples collected in the pandemic (2020) and post-pandemic (2022) periods

	2020			2022		
	Range	Median	Frequency of detection (%)	Range	Median	Frequency of detection (%)
PM_2.5_ (µg m^−3^)	5–35	17	--	6–71	24	--
Carbonaceous species (µg m^−3^)						
OC	0.5–16.8	3.5	100	1.3–16.8	4.8	100
EC	0.06–3.6	0.7	100	0.1–4.2	1.1	100
WSI (ng m^−3^)						
F^−^	25–230	53	98	2–190	42	89
Formate	111–152	127	47	18–177	68	95
Cl^−^	11–729	156	80	10–1818	157	95
NO_3_^−^	134–2340	891	100	219–4206	976	100
SO_4_^2−^	232–4260	1462	100	436–6682	1904	100
Oxalate	29–152	86	90	28–685	157	100
Na^+^	12–255	88	86	50–776	179	100
NH_4_^+^	17–1141	338	100	82–2186	576	100
K^+^	15–520	146	37	28–776	204	100
nss-K^+^	25–515	143	37	1–497	175	100
Ca^2+^	35–374	113	96	3–95	19	95
Mg^2+^	15–190	56	57	1–190	42	95

No significant differences were observed in meteorological parameters (precipitation, relative humidity, temperature, solar radiation, and wind speed) between the 2 years (*p* > *0.05*)*.* The Spearman analysis revealed only weak to moderate correlation of PM_2.5_ with meteorological variables in both years. PM_2.5_ correlated negatively with relative humidity (RH, *ρ* = −0.4), wind speed (WS, *ρ* = −0.4), and precipitation (Ppt, *ρ* = −0.3) in 2020 (Table S7). In 2022, PM_2.5_ showed a moderate negative correlation with Ppt (*ρ* = −0.4) (Table S8).

The atmospheric boundary layer (ABL) is a key factor in dispersing atmospheric pollutants. In São Paulo, the ABL height is usually lower in colder seasons (Moreira et al. 2024), and sea breezes can further reduce the boundary layer height in both warm and cold seasons (Ribeiro et al. 2018). A study conducted between 2018 and 2021 in the metropolitan region of São Paulo found that the seasonal patterns and the influence of meteorological variables remained consistent during this period: summer offered the best conditions for pollutant dispersion, whereas winter had less favorable conditions (lower ventilation coefficient and lower atmospheric boundary layer height). Therefore, the main factor behind the reduction in PM was the decline in vehicular traffic (Moreira et al. 2023).

### Source apportionment by factor analysis-multiple linear regression (FA-MLR)

The source apportionment of PM_2.5_ was conducted by FA-MLR analysis. Three factors were extracted and explained more than 60% of the variance in the data (Table S10). Multiple linear regression (MLR) analysis of the unnormalized factor scores as a function of PM_2.5_ concentrations was performed to assess the contributions of sources. All regression coefficients were significant (*p* < 0.0001), and the adjusted R^2^ values were 0.89 in 2020 and 0.87 in 2022 (Tables S11–S13). 

The comparison between observed and modeled PM_2.5_ concentrations over time is shown in supplementary information (Fig. S4 and S5). The results calculated with the MLR coefficients reconstructed the PM_2.5_ satisfactorily. The contributions from unaccounted sources were 23% in 2020 and 9% in 2022. Therefore, the sources explained more than 75% of PM_2.5_ in both years.

**Fig. 1:**
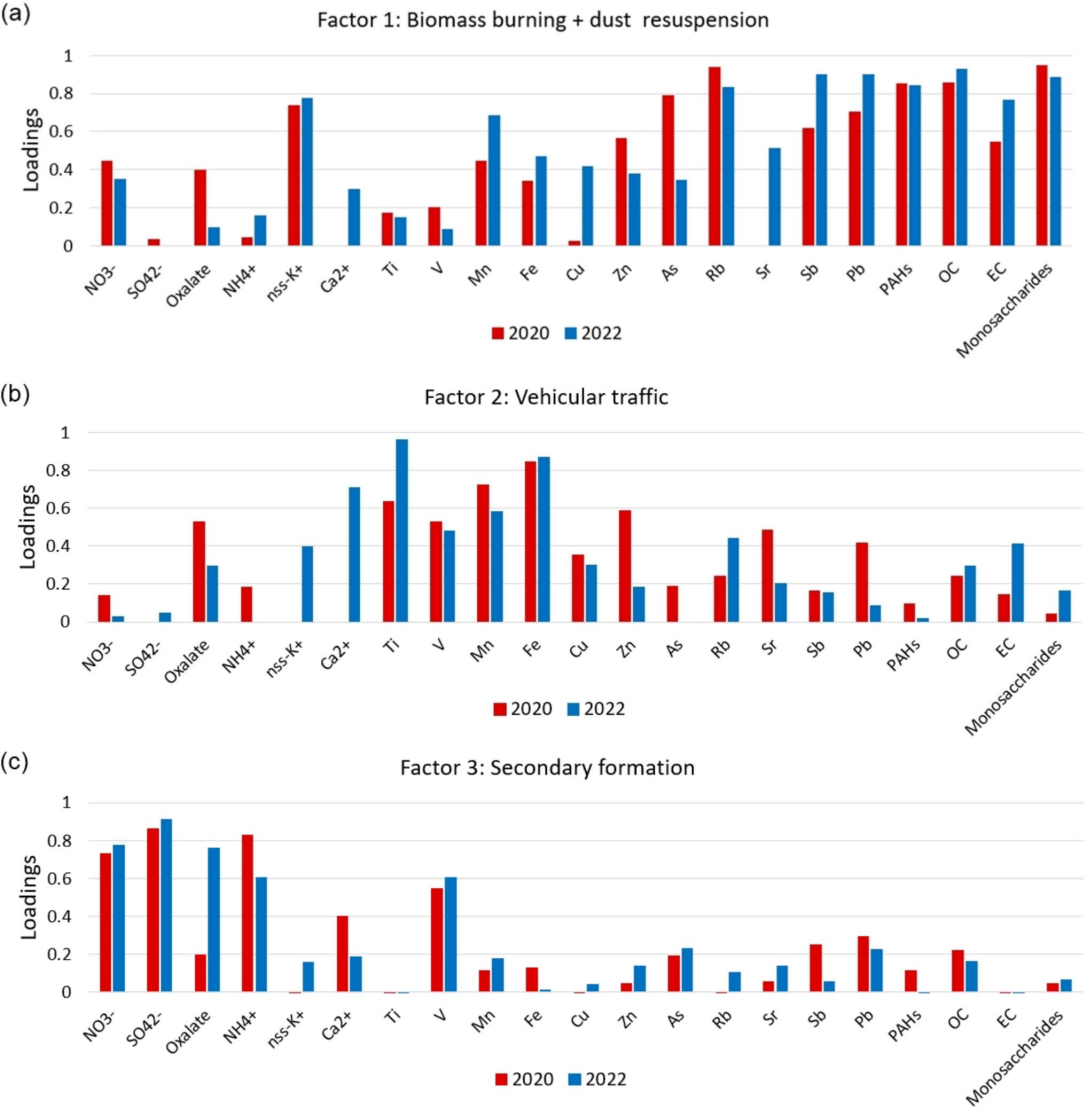
PM_2.5_ source profiles obtained by factor analysis: **a** Biomass burning and dust resuspension, **b** vehicular traffic, and **c** secondary formation

The factor profiles are presented in Fig. 1. The sources identified were biomass burning and dust resuspension, vehicular traffic, and secondary formation.

Factor 1 presented high loadings (>0.6) of biomass burning markers (monosaccharides, nss-K^+^, and Rb) (Simoneit et al. 2004; Pachon et al. 2013; Trieu et al. 2024), PAHs, OC, EC, As, Pb, and Sb, in addition to moderate loadings of elements that may be related to dust resuspension during burning events (Fe, Mn, and Zn). The enrichment of metals (such as Zn, As, Pb, and Sb) has been reported in ashes and PM samples collected from wildfires, waste, coal, and wood combustion (Liu et al. 2025; Rao and Parsai 2025). In Brazil, the enrichment of Pb in PM_2.5_ samples collected during wildfires in northern Pantanal was attributed to soil contamination by mining activities (Ramos et al. 2024). In a study conducted at this site, Pereira and collaborators (2025) reported the presence of Sb, Pb, and Cd in the biomass burning profile and suggested the influence of waste incineration or mixture with vehicular traffic sources (Pereira et al. 2025). Biomass burning and soil dust resuspension contributed to 36% and 34% of the PM_2.5_ concentrations in 2020 and 2022, respectively.

Sources of biomass burning inside and outside of the Metropolitan Area of São Paulo (MASP) contribute to air pollution in the city (Kumar et al. 2016). Within the city, sources include pizzerias, bakeries, and steakhouses. There are over 8000 pizzerias in the city, and about 80% of them use biomass fuelwood ovens (eucalyptus hardwood and briquettes) (Sgarbi et al. 2013; Lima et al. 2020). Despite social restrictions during the pandemic, many food establishments continued operating with delivery services, and pizzeria operations increased during this period compared to previous years (APUBRA 2022).

Outside sources include forest fires and agricultural burning. In 2020, the National Institute for Space Research (INPE) reported more than 2400 fires in São Paulo state during the studied period (INPE 2020). In 2022, more than 930 fires were reported in the same period (INPE 2022). It is important to note that plumes coming from the Amazon forest can cross the country and arrive in the southern areas of Brazil (Souto-Oliveira et al. 2023; Pereira et al. 2021).

Factor 2 was characterized by high loadings of elements associated with dust resuspension (Fe, Ti, and Mn), as well as weak to moderate loadings of species related to vehicular exhaust and non-exhaust emissions (Cu, Zn, V, Pb, NO_3_^−^, oxalate, OC, and EC) (Pereira et al. 2023). The mixed profile of vehicular emissions and dust resuspension was already observed in a previous study carried out at this site, where the source apportionment was realized by Positive Matrix Factorization (PMF) (Pereira et al. 2025). This factor was attributed to vehicular traffic and contributed to 30 and 35% of PM_2.5_ concentrations observed in 2020 and 2022, respectively.

Copper presented medium loadings in this factor in both years (>0.30). This element is a vehicular traffic marker, associated with exhaustive and non-exhaustive vehicle emissions (Grigoratos and Martini 2015; Pereira et al. 2023). The presence of copper at this site is mainly attributed to emissions from ethanol and gasohol-fueled vehicles, due to ethanol processing in copper tanks and engine corrosion (Pereira et al. 2023; Sánchez-Ccoyllo et al. 2009).

Factor 3 showed high loadings of sulfate, nitrate, and ammonium (SNA), in addition to some contribution of oxalate and OC, which indicates secondary formation. The SNA ions are formed in the photooxidation of gaseous pollutants (SO_2_, NO_x,_ and NH_3_, respectively) (Manahan 2009). Vehicular traffic is the most important source of these pollutants in urban sites, with a greater contribution from diesel-powered vehicles (Chang et al. 2019; Yasar et al. 2013; Degraeuwe et al. 2016). This source contributed to 11% and 23% of PM_2.5_ concentrations during and after the COVID-19 pandemic.

Overall, the source’s profiles (Fig. 1) obtained by factor analysis were similar in the two periods, and the slight differences observed in the profiles of biomass burning and vehicular traffic may reflect changes in the types of biomass burned and in the use of vehicular fuels (mainly ethanol and gasohol). It is important to note that the small number of samples collected (*n* = 93) in these periods may have limited the observation of sources with smaller contributions, such as industrial emissions, which could overestimate the contribution of some sources. In fact, the contribution of industrial emissions is less observed, due to smaller contributions and the variability of processes and activities involved (Lucarelli et al. 2020; Fadel et al. 2024). Yet, the FA-MLR analysis allowed the identification of the most important sources and reconstruction of PM_2.5_ mass.

### Carbonaceous species

The concentrations of elemental carbon (EC) and organic carbon (OC) (Table 1) were higher in 2022 than in 2020, but no significant differences were observed for OC/EC ratios (*p ˃ 0.05*), which presented a median value of 4.8 (from 1 to 17) in 2020 and 4.4 in 2022 (from 2 to 11). The reduction in EC concentrations during the restriction measures period was also reported in sites impacted by vehicular traffic and industrial emissions in Jiangsu province, China (Jia et al. 2021), and Jamshedpur, India (Ambade et al. 2021). Conversely, some studies reported increased ozone levels in urban sites, leading to higher atmospheric oxidative capacity and increased secondary organic aerosol formation during this period (Sun et al. 2020; Xu et al. 2022).

OC/EC ratios lower than 2 are commonly observed in urban areas affected by vehicular traffic, while higher ratios suggest secondary organic carbon formation and/or the impact of biomass burning (Pereira et al. 2025). OC and EC concentrations presented a strong correlation with anhydride monosaccharides (*ρ* > 0.70) and moderate to strong correlations with PAHs *(*ρ = 0.33–0.87) (Tables S7 and S8) in both years. The increase in the OC/EC ratio had already been observed in São Paulo samples and was attributed to the increased use of biofuels and improvements in combustion efficiency in vehicle engines (Pereira et al. 2025), yet the results of the present study indicate that biomass burning may be contributing to the observed concentrations. In fact, the source apportionment showed that these pollutants were mainly associated with biomass burning. 

### Water-soluble ions

Water-soluble ions (WSIs) concentrations are presented in Table 1. Most ion levels increased significantly in 2022 (*p* < *0.05*), except for calcium, magnesium, formate, and fluoride. Sulfate, nitrate, and ammonium (SNA) were the most abundant ions in both years. These species are formed in the photooxidation of gaseous pollutants (SO_2_, NO_x_, and NH_3_, respectively) (Manahan 2009), and the rise in concentrations after the pandemic was expected.

In 2020, WSI showed lower concentrations than those seen in previous studies at this site, when the concentrations of SO_4_^2−^, NO_3_^−^, and NH_4_^+^ were nearly twice as high as those observed in this study (Pereira et al. 2025; Serafeim et al. 2023). Interestingly, after the pandemic, nitrate and ammonium concentrations decreased compared to the pre-pandemic studies (Pereira et al. 2025). Conversely, the concentrations of sulfate were higher in 2022 (15%). The reduction of nitrogen ionic species may reflect the trend of NO_2_ reduction in São Paulo state observed in recent years due to previous changes in the legislation (Girotti et al. 2024).

The median NO_3_^−^/SO_4_^2−^ was 0.6 in both years. It is reported that NO_3_^−^/SO_4_^2−^ ratios smaller than 1 indicate the dominance of stationary sources (e.g., industrial emissions), while ratios greater than 1 indicate the main contribution of mobile sources (Huang et al. 2016). However, NO_3_^−^ volatilization can cause losses of this PM_2.5_ component (Babich et al. 2000). [NH_4_^+^]/[NO_3_^−^] and [NH_4_^+^]/[SO_4_^2−^] ratios were 1.2 and 1.0 in 2020, and 2.4 and 2.0 in 2022, respectively, which indicates the predominance of ammonium nitrate (Kuniyal et al. 2015). [NH_4_^+^]/[SO_4_^2−^] ratios smaller than 2 also indicate the possible association and neutralization of SO_4_^2−^ by cations emitted through mineral dust resuspension (e.g., Ca^2+^ and Mg^2+^) (Ram et al. 2012). In 2020, calcium showed moderate positive correlations with the SNA (*ρ* = 0.6–0.7) (Table S7).

Organic ions (oxalate and formate) can be produced through secondary reactions and released directly from biomass burning, fossil fuel combustion, and biogenic emissions (Tsai et al. 2013). Unlike the present study, some researchers have reported the increase of oxalate and its precursors (e.g., oxalic and glyoxal) during the lockdown, attributed to higher atmospheric oxidation capacity (Meng et al. 2021; Chen et al. 2023). In São Paulo, the reliance of precursors emitted by human activities, such as vehicular emissions, may have limited the concentrations observed during the COVID-19 pandemic.

The Cl^−^/Na^+^ ratios were below the value attributed to marine aerosol (Cl^−^/Na^+^ = 1.8, Fu et al. 2004) in both years (1.3 in 2020 and 0.9 in 2022). The lower value observed in 2022 indicates a greater chloride depletion that year. The reaction of chloride with an acidic species (e.g., nitric or sulfuric acid) is a primary depletion pathway, leading to the formation of HCl. The evaporation of hydrochloric acid leads to the loss of chloride in the particulate matter (Su et al. 2022).

The contribution of non-sea-salt potassium (nss-K^+^, calculated as described by George et al. 2008, Supplementary Information) corresponded to more than 90% of total potassium, indicating the predominance of other sources besides marine aerosol, such as biomass burning and dust resuspension (Zhang et al. 2013; Pachon et al. 2013). The concentrations of potassium in 2020 were also lower than those observed during the wintertime (June to September) in 2019 (247 ng m^−3^; Pereira et al. 2025). Nevertheless, the concentrations observed during the lockdown in 2020 were slightly higher than those observed in a campaign carried out in the period before the lockdown (July to December 2019) (110 ng m.^−3^, Serafeim et al. 2023). The reduction in concentrations of this ion in PM_2.5_ during the COVID-19 outbreak was also observed in urban areas in Suzhou (China) and Elche (Spain) (Wang et al. 2021a; Clemente et al. 2022), and although biomass burning is an important source of potassium in the atmosphere, the reduction in vehicular traffic and dust resuspension may also have influenced this result.

### Determination of trace elements

The concentrations of the elements are presented in Table 2. Fe, Cu, and Zn were the most prevalent species in both years. Ti, Cr, Mn, Fe, Ni, Zn, As, Sr, and Ba (*p* < *0.05*) exhibited a significant increase in 2022. The higher concentrations observed after the pandemic period indicate an increase in emissions from human activities, such as exhaust and non-exhaust vehicular emissions, dust resuspension, and industrial emissions. V, Se, Rb, Cd, Sn, Sb, and Pb did not present significant changes in their concentrations between these periods.

**Table 2:** Trace elements in PM_2.5_ samples collected during restrictive measures in São Paulo during (2020) and the post-pandemic period (2022)

Elements (ng m^−3^)	2020 campaign			2022 campaign		
	Range	Median	Frequency of detection (%)	Range	Median	Frequency of detection (%)
Ti	0.1–10.1	2.2	82	0.3–45.0	6.4	98
V	0.05–2.00	0.42	100	0.04–1.87	0.62	98
Cr	0.06–3.10	0.53	57	0.05–3.91	1.33	70
Mn	0.3–8.6	2.4	98	0.5–17.8	6.4	100
Fe	4–205	45	100	11–833	191	98
Ni	0.04–5.00	0.35	73	0.2–1.7	0.7	98
Cu	31–1056	95	100	10–99	32	100
Zn	2.5–80	27.2	100	6–295	50	100
As	0.04–0.95	0.34	100	0.07–1.25	0.51	73
Se	0.03–3.77	0.31	94	0.07–2.93	0.52	68
Rb	0.04–4.20	0.89	100	0.1–3.6	1.0	100
Sr	0.05–1.86	0.62	76	0.2–13.8	1.2	95
Mo	0.2–167.0	5.3	100	0.6–8.4	2.1	100
Cd	0.02–1.10	0.16	100	0.05–2.30	0.27	84
Sn	0.1–15.0	2.0	100	0.2–29.6	3.4	100
Sb	0.4–17.0	2.4	100	0.4–18.6	2.9	100
Ba	0.1–47.0	1.8	86	1–35	8	86
Pb	0.6–18.0	5.7	100	1–26	7	100

Unexpectedly, Cu and Mo showed a significant decrease (*p* < *0.05*) after the economic reopening, with concentrations lower than those observed during the pandemic and reported in previous studies. In the winter of 2019, concentration ranges were 13–931 ng m^−3^ for Cu and 0.1–31.2 ng m^−3^ for Mo (Pereira et al. 2025). Cu and Mo exhibited moderate (*ρ* = 0.59) and strong (*ρ* = 0.83) correlation in 2020 and 2022, respectively, indicating similar emission sources. As mentioned earlier, copper is associated with emissions from ethanol and gasohol-fueled vehicles (Pereira et al. 2023; Sánchez-Ccoyllo et al. 2009). Mo is presented as a catalytic converter in ethanol-fueled vehicles (La Colla et al. 2021), as an additive in lubricant oil, and in brake linings (Smichowski et al. 2008; Alves et al. 2015).

The Brazilian vehicular fleet is mainly fueled by ethanol (hydrated ethanol) and gasohol (gasoline C), which is a blend of 27% anhydrous ethanol and 73% pure gasoline (gasoline A). In 2022, the lower price competitiveness of hydrated ethanol compared to gasoline C led to a decline in its sales. Compared to 2020, hydrated ethanol sales in São Paulo dropped by 21.6% this year (ANP 2023). Although LDV traffic decreased by 55% during the first months of the pandemic (Pérez-Martínez et al. 2022), the higher ethanol consumption in 2020 is likely the main reason for the concentrations observed for Cu and Mo.

Among the metals quantified in this study, Fe is the most abundant in the crust (Lee 1999) and has already been associated with soil resuspension in previous studies due to an enrichment factor (EF) lower than 10 compared to Al (Pereira et al. 2025). In this study, Fe was used as a reference element for the EF calculation.

**Fig. 2:**
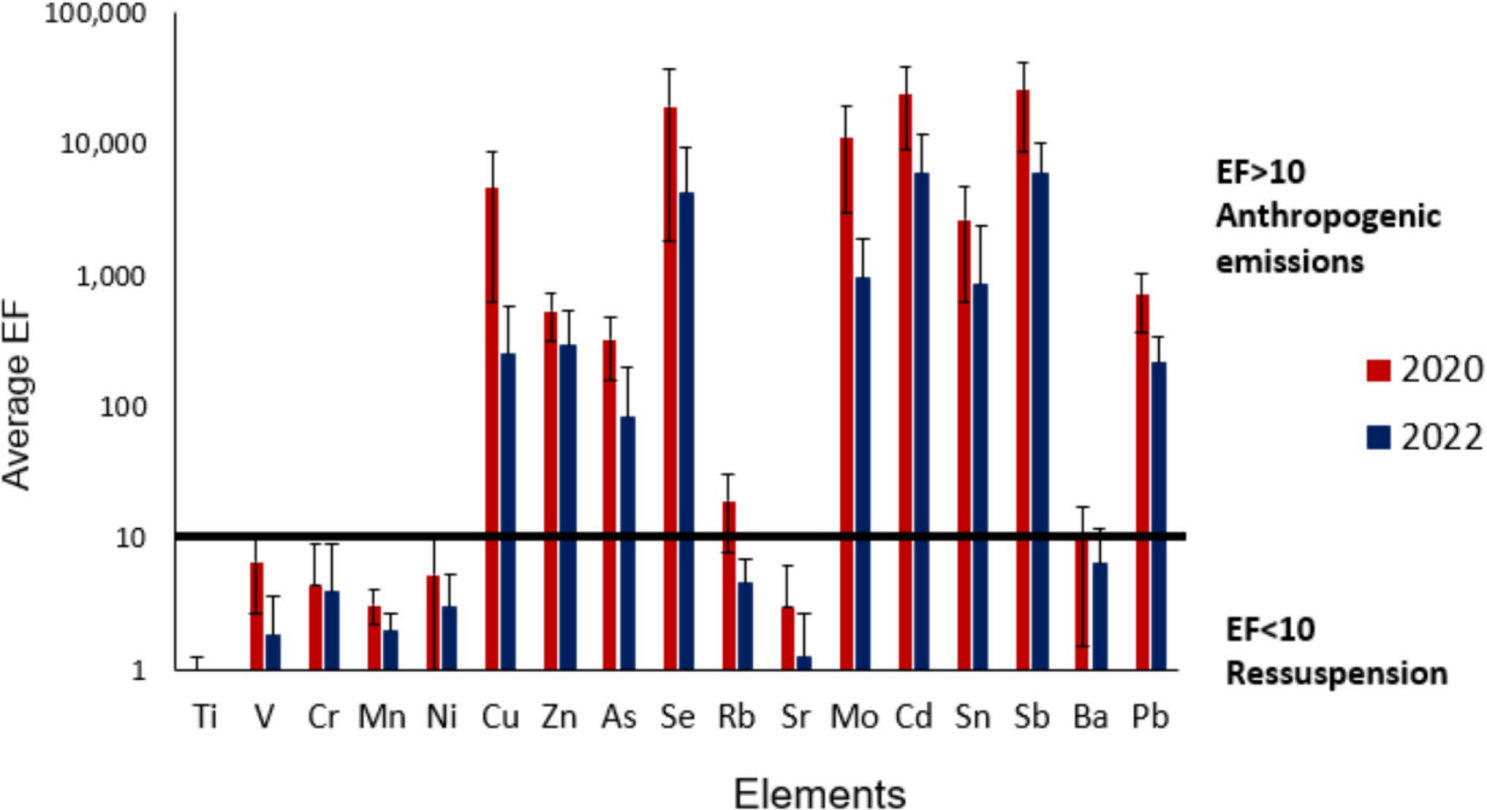
Enrichment factors (EF) of elements in PM_2.5_ samples collected in São Paulo during (2020) and after (2022) the COVID-19 pandemic

Particulate matter is considered enriched with elements when the calculated EF exceeds 10, while an EF below 10 indicates no contributions from sources other than soil resuspension (Gelado-Caballero et al. 2012; Shelley et al. 2015). The enrichment profile (Fig. 2) remained consistent between the periods studied, except for Rb, which showed an enrichment in 2020, and EF smaller than 10 in 2022. V, Ti, Cr, Mn, Ni, Ba, and Sr had EFs less than 10 in both years, suggesting that soil dust resuspension might be the primary source. Zn, Cu, Pb, Mo, As, Se, Sn, Cd, and Sb were enriched relative to Fe, indicating that anthropogenic sources contributed to the emissions of these chemical species during and after the pandemic period.

Although the element concentrations were higher for most species in 2022, the EF values were greater during the pandemic. This is due to an increase in dust resuspension in 2022, driven by higher vehicular traffic, industrial, and construction activities.

Se, Cd, and Sb exhibited higher EF values in both years (EF > 1000). Other studies identify various sources for these elements. The element Se in urban atmospheric samples mainly comes from industrial emissions and fossil fuel combustion (Duan et al. 2012; Etteieb et al. 2020; Huang et al. 2022). Industrial emissions and residential coal burning were previously identified as sources of Se in the particulate matter collected in the São Paulo metropolitan area (Miranda et al. 2018). In this Brazilian city, industrial emissions often contribute to a smaller portion of PM_2.5_ (~4–16%) (Miranda et al. 2018; Serafeim et al. 2023; Pereira et al. 2025). Lima and collaborators (2020) found high levels of selenium in PM_2.5_ samples collected from pizzeria chimneys in São Paulo.

Notably, Sb was associated primarily with biomass burning in the present study. The association of Sb and Cd with biomass burning and waste incineration was discussed previously, but these elements may also be related to vehicular traffic. Sb is mainly linked with brake wear emissions (Fomba et al. 2018). Additionally, Nory and collaborators (2021) found an enrichment of Cd, Sb, and Cu in dust particles collected in a tunnel affected by light-duty vehicles in São Paulo. These elements were associated with brake wear. In another study conducted in Brazilian tunnels, Pereira and collaborators (2023) concluded that, due to Cu emissions from ethanol and gasohol, Sb, Ba, and Zn serve as more representative markers for brake abrasion emissions than Cu. Road dust, brake, and tire wear were also reported as the major sources of Cd in urban sites (Silva et al. 2016; Jandacka et al. 2017; Vannini et al. 2019; Ramírez et al. 2020).

Overall, it was observed that most elements showed moderate to strong correlations in both years. In 2020, there were strong correlations between Fe, Mn, Zn, and Pb (*ρ* > 0.70). These elements also showed moderate to strong correlations (*ρ* > 0.40) with Ti, As, Sn, Rb, Cd, and Sb. In 2022, the crustal elements Fe, Mn, Ti, and Sr had strong correlations (*ρ* > 0.70). These species also exhibited moderate to strong correlations with Rb, Ba, Sb, Pb, As, and Cd (*ρ* > 0.50). These findings suggest the influence of different sources, such as dust resuspension and vehicular traffic emissions (including exhaust and non-exhaust emissions).

Rb also exhibited strong correlations with anhydride monosaccharides (levoglucosan, mannosan, and galactosan) in both years (*ρ* > 0.80), along with moderate (*ρ* > 0.60) and strong (*ρ* > 0.90) correlations with potassium in 2020 and 2022, respectively. Previous studies reported the association of Rb with biomass burning and soil resuspension (Trieu et al. 2024), which is also consistent with the correlation observed with crustal elements (Fe, Mn, Zn, and Ti).

### Monosaccharides

The Mann–Whitney test revealed no significant differences between the concentrations of anhydride monosaccharides (levoglucosan, mannosan, and galactosan) during (2020) and after the COVID-19 pandemic (2022) (Table 3), indicating that there was no change in biomass burning emissions during this period.

**Table 3:** Concentrations and diagnostic ratios of monosaccharides in the PM_2.5_ samples collected in São Paulo during (2020) and post-pandemic (2022)

Monosaccharides (ng m^−3^)	2020			2022			Mann–Whitney
	Range	Median	Freq. of det.(%)	Range	Median	Freq. of det. (%)	W rank-sum statistic
Levoglucosan	5–615	147	100	30–870	165	98	813
Mannosan	2–76	16	94	2–86	13	98	939
Galactosan	2–31	8	96	2–55	10	91	726
L/M	1–14	10	--	5–85	12	--	--
L/G	3–41	20	--	11–52	18	--	--
Fucose	2–6	2	14	1–10	2	20	19
Arabinose	2–5	2	63	1–26	3	91	560
Galactose	2–11	3	55	2–29	5	87	220***
Rhamnose	7–81	38	6	--	--	--	--
Glucose	2–152	38	73	4–2523	69	96	464**
Xylose	2–50	10	45	1–103	23	78	154***
Mannose	2–37	9	35	2–102	4	67	245

The Mann–Whitney−Wilcoxon rank−sum statistic, W, indicates whether the sum of the ranks for the observations of the first group (2020) is different than that expected under the null−hypothesis

**p < *0.01* and ***p < *0.001 *indicate significant difference

The ratios between the concentrations of levoglucosan and its isomers (galactosan and mannosan) were calculated. L/M ratios lower than 10 are often linked to softwood combustion, while higher values (~14–26) suggest hardwood and crop residues burning (Theodosi et al. 2018; Bhattarai et al. 2019; Gonçalves et al. 2021). In Brazil, L/M ratios near 10 were found in some agroindustrial regions, where the emissions are highly influenced by sugarcane straw burning (Hall et al. 2012). These median values were close to 10 in both campaigns, but there was more variation in 2022. That year, L/M values were close to the range observed in the winter campaign of 2019 (L/M: 6.7–58.0, Pereira et al. 2025). Although sugarcane burning has decreased due to bans, fires related to agriculture, criminal or natural causes are still observed in the interior of the state (Scaramboni et al. 2024). Burning of bagasse to produce energy in sugarcane refineries and power plants may also influence these values (CONAB 2011). Schmidl and collaborators (2008) suggested that low L/G ratios (<2) may be linked to leaf burning, while values higher indicate wood burning. In this study, the L/G ratio presented a large variation in both years, which suggests the burning of various biomass types.

**Fig. 3:**
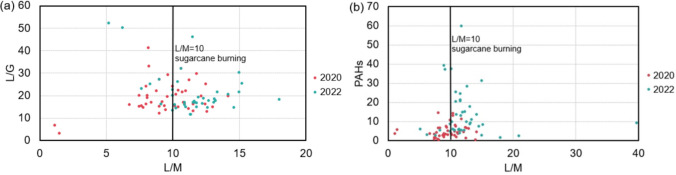
Anhydride monosaccharides ratios: **a** L/G vs. L/M and **b** sum of PAH vs L/M

Figure 3a displays the L/M vs. L/G plot. The L/M ratio varied between 8 and 12 in 78% of PM_2.5_ samples collected in the pandemic (2020) and 61% of samples collected after economic reopening (2022). In 2022, the samples were more likely to have L/M ratios greater than 10. The L/G ratio mostly ranged between 10 and 30 in both years. Figure 3b shows the sum of PAHs vs. L/M ratio plot. Notably, PAH concentrations tend to increase with the L/M ratio between 10 and 15 in the 2022 samples, indicating that biomass burning contributed more to the concentrations of these pollutants during those days. These results highlight a greater contribution from hardwood burning in 2022.

**Fig. 4:**
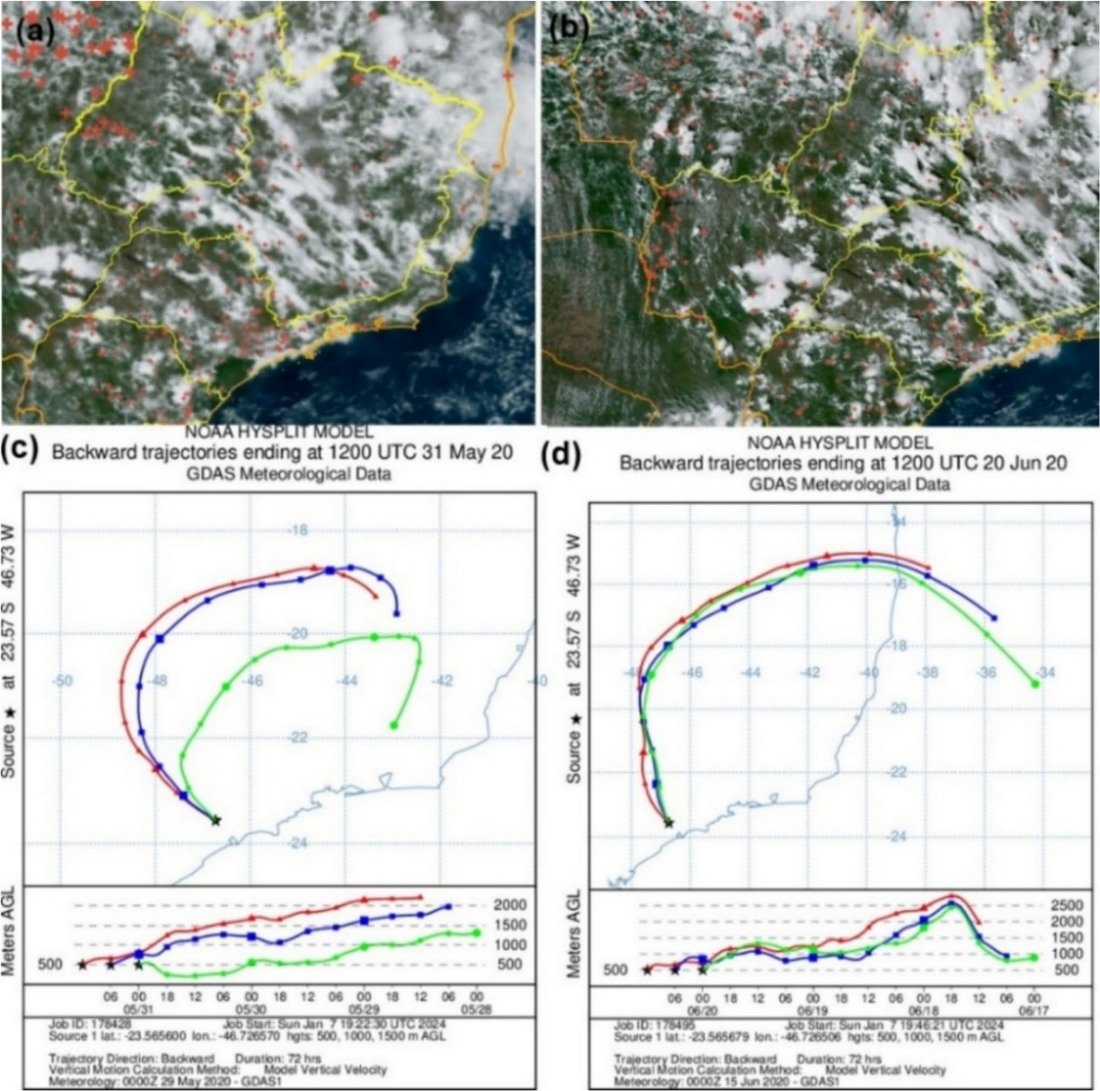
Fire spots reported by INPE (National Institute for Space Research) data and Hysplit backward trajectories on days with peak concentrations of anhydrides monosaccharides: **a** Fire spots detected from May 27th to 31 st 2020 (**b**) Fire spots detected from June 16th to 20th 2020. **c** Backward air masses trajectories on May 31 st 2020 and **d** backward air masses trajectories on June 20th 2020

**Fig. 5:**
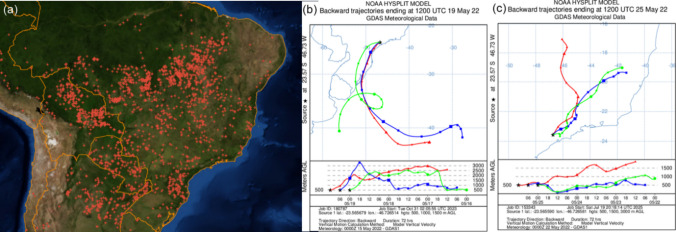
Fire spots reported by INPE (National Institute for Space Research) data and Hysplit backward trajectories on days with peak concentrations of retene and levoglucosan in 2022: **a** Fire spots detected from May 16th to 25th 2022. **b** Backward air masses trajectories on May 19th 2022 and **c** May 25th 2022

The Hysplit model and INPE data showed that on days with high concentrations of monosaccharides, the air masses passed over burning regions in Minas Gerais and in the interior of the São Paulo state (Fig. 4), in Southern Brazil (Fig. 5b), in Central West, Minas Gerais, and in the interior of São Paulo state (Fig. 5c). This shows the contribution of the transport of air masses from regions with forest fires and agro-industrial burning.

No significant differences were observed in most primary monosaccharides (mannose, galactose, fucose, and arabinose) between the two periods, except for glucose and xylose, which showed concentrations significantly higher in 2022 (*p* < *0.05*). Primary monosaccharides are present in structural components of plants, fungi, and bacteria. These compounds can be emitted directly from biogenic particles such as pollen, leaves, and spores, or released during biomass burning and soil dust resuspension (Tominaga et al. 2011; Simoneit et al. 2004).

Glucose was the most abundant in both years, followed by xylose and mannose. These sugars are components of cellulose, xylans, and mannans from plant cell walls (Schädel et al. 2010), and were also present in high abundance in the particles of the Black Rain event in the São Paulo metropolitan area (Pereira et al. 2021). Besides levoglucosan, glucose is often found in abundance in airborne samples (Theodosi et al. 2018; Marynowski and Simoneit 2022). Some authors have also suggested that glucose could serve as a pollen grain tracer (Marynowski and Simoneit 2022).

### Polycyclic aromatic hydrocarbons

The concentrations of individual and total PAHs were significantly lower during the pandemic (2020) (*p* < *0.001*), except for retene (RET) and indeno(1,2,3-c,d)pyrene (INP), which did not display significant changes in concentrations (Table 4). The total PAHs concentrations observed in 2020 (∑PAHs = 0.2–14.6 ng m^−3^) were ~2–3 times lower than those reported in the winter of the previous year (∑PAHs = 1.1–37.3 ng m^−3^) (Pereira et al. 2025). After the pandemic (2022), the concentrations reached levels close to or even surpassing those recorded before the pandemic (∑PAHs = 1.6–60.0 ng m^−3^).

**Table 4:** Concentrations of PAHs and derivatives (oxy and nitro-PAHs) in PM_2.5_ samples collected in São Paulo during (2020) and post-pandemic (2022)

Conc. (ng m^−3^)		2020			2022		
	Abbreviation	Range	Median	Freq. of det. (%)	Range	Median	Freq. of det. (%)
PAHs							
Phenanthrene	PHE	0.01–1.50	0.06	82	0.01–0.45	0.09	93
Fluoranthene	FLT	0.02–0.78	0.09	90	0.04–1.40	0.24	100
Pyrene	PYR	0.03–0.74	0.11	90	0.06–1.05	0.23	100
Retene	RET	0.02–0.54	0.07	41	0.02–0.38	0.11	82
Benzo(a)anthracene	BaA	0.02–0.56	0.09	94	0.03–2.00	0.25	100
Chrysene	CHR	0.03–0.88	0.15	98	0.10–2.60	0.47	100
Benzo(b)fluoranthene	BbF	0.07–3.34	0.72	100	0.1–13.8	1.18	100
Benzo(k)fluoranthene	BkF	0.04–0.77	0.18	90	0.07–4.50	0.48	100
Benzo(e)pyrene	BeP	0.03–1.48	0.33	96	0.2–11.6	1.10	100
Benzo(a)pyrene	BaP	0.02–1.15	0.23	86	0.07–5.23	0.59	100
Perylene	PER	0.02–0.22	0.11	22	0.01–1.81	0.26	73
Indeno(1,2,3-c,d)pyrene	INP	0.07–3.02	0.77	98	0.1–6.4	0.87	100
Dibenzo(a,h)anthracene	DBA	0.06–0.59	0.14	27	0.10–5.12	0.71	93
Benzo(g,h,i)perylene	BPE	0.05–2.37	0.62	98	0.2–5.4	1.31	100
Coronene	COR	0.06–1.30	0.29	84	0.07–6.87	0.65	100
	∑PAHs	0.24–14.60	3.60		1.6–60.0	8.8	
Oxy-PAHs							
9-Fluorenone	9-FLO	0.02–0.07	0.03	29	0.03–2.13	0.26	93
9,10-Anthraquinone	9,10-AQ	0.02–1.90	0.14	71	0.20–2.20	0.60	93
2-Methylanthraquinone	2-MAQ	0.04–0.45	0.15	45	0.06–2.60	0.66	93
7,12-Benzo(a)anthracedione	7,12-BaAQ	0.04–1.10	0.25	55	0.23–4.50	0.50	48
	∑Oxy-PAHs	0.04–3.10	0.37		0.01–6.50	1.90	
Nitro-PAHs							
9-Nitroanthracene	9-NANT	0.01–0.02	0.02	6	0.02–0.41	0.11	43
2 + 3-Nitrofluoranthene	2 + 3-NFLT	0.05–0.40	0.18	22	0.01–0.84	0.07	64
1-Nitropyrene	1-NPYR	0.03–0.36	0.16	22	0.01–0.20	0.08	34
6-Nitrochrysene	6-NCHR	0.08–0.09	0.09	2	0.01–0.19	0.01	27
	∑Nitro-PAHs	0.02–0.63	0.26		0.01–1.50	0.16	

The economic reopening was the main cause of the increase in PAHs concentrations in 2022, but the changes in fuel consumption patterns also made a significant contribution. Contrary to the decrease in ethanol consumption, the gasoline sales in São Paulo state increased by 40% in 2022 in comparison with 2020, and by 27% compared to 2019 (ANP 2023). The literature indicates that gasohol emits more PAHs than hydrated ethanol combustion (Pereira et al. 2023; Abrantes et al. 2009). The sales of diesel presented a smaller variation, increasing by 1.3% and 4% compared to 2019 and 2020, respectively (ANP 2023).

The most abundant compounds were BbF (22%), INP (20%), and BPE (15%) in 2020, and BbF (15%), BeP (14%), and BPE (13%) in 2022. High molecular weight PAHs (HMW-PAHs, five or more rings) were dominant in both years, accounting for 83% of total PAHs in 2020 and 92% in 2022. Despite the higher vapor pressures favoring the distribution of low molecular PAHs (LMW-PAHs, 2 or 3 rings) in the gaseous phase (Dat and Chang 2017), the predominance of HMW-PAHs may be associated with combustion processes; in urban sites, vehicular emissions and some industrial processes are the main sources (Thang et al. 2019; Cao et al. 2021; Tian et al. 2021).

Vehicle emissions are often recognized as major sources of HMW-PAHs (Tian et al. 2021). Pyrogenic PAHs (BbF, BPE, and INP) are linked with vehicle exhaust emissions (Romagnoli et al. 2019) and were the most abundant PAHs in a tunnel affected by emissions from gasoline-powered vehicles in Nanjing (China) (Fang et al. 2019). BPE is regarded as a marker of gasoline emissions, and the connection between CHR and vehicular traffic is also noted in the literature (Mellado et al. 2022). COR, an HMW-PAH and vehicular emissions marker (Pereira et al. 2023), was detected on 84% and 100% of campaign days in 2020 and 2022, respectively. In a recent study carried out in tunnels influenced by light-duty vehicles in São Paulo (Pereira et al. 2023), PAHs with four and five rings (CHR, BeP, BaP, and BPE) were the most prevalent.

The predominance of HMW-PAHs had already been observed in earlier studies conducted at this site. In 2002, Bourotte et al. (2005) reported the highest abundance of INP (23%) in PM_2.5_, followed by BPE (22%) and BbF (11.4%). In the campaigns conducted in 2019, BbF was the most abundant PAH. INP, BPE, and BeP were also abundant (Pereira et al. 2025, Serafeim et al. 2023).

**Fig. 6:**
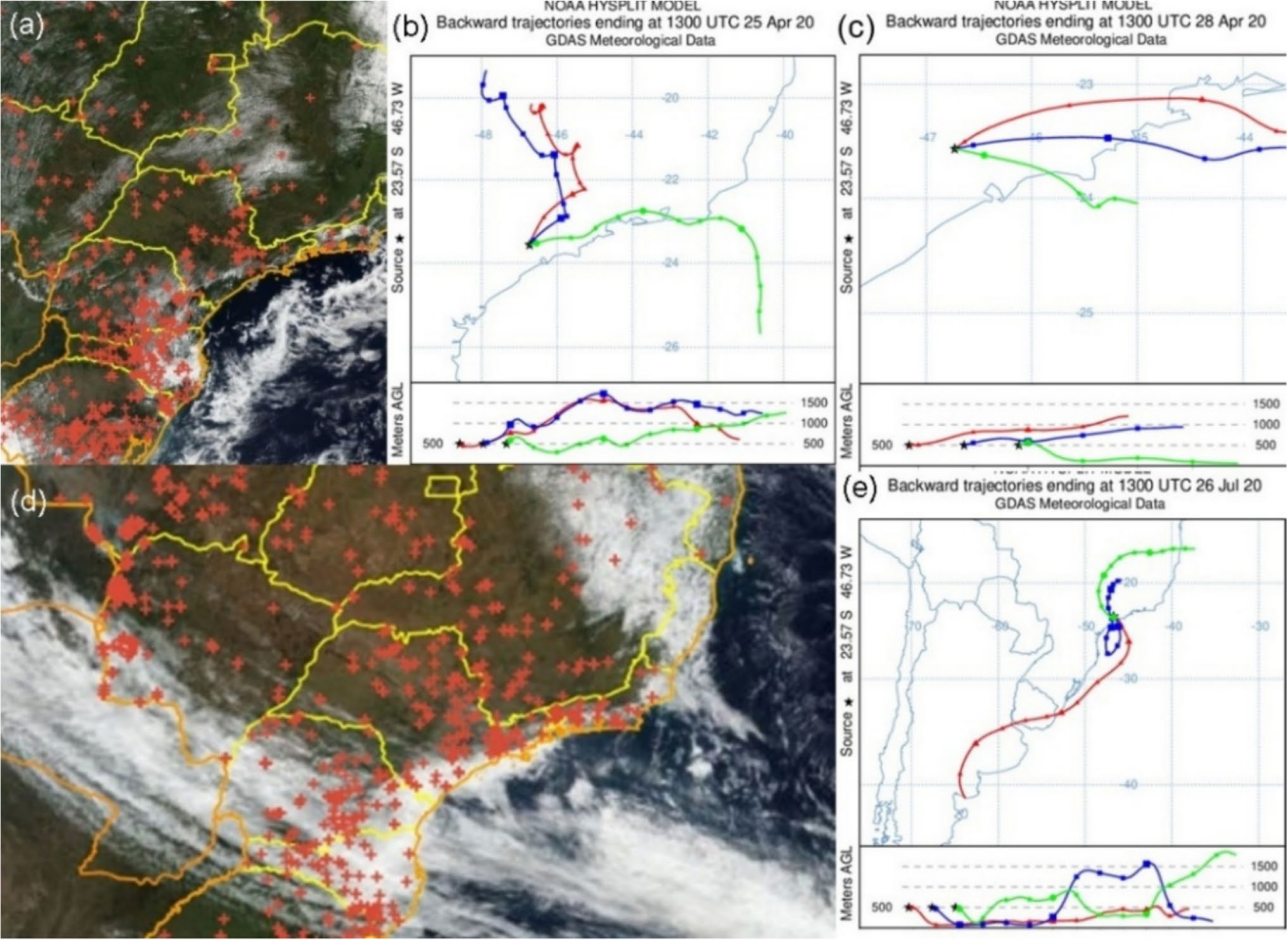
Fire spots reported by INPE (National Institute for Space Research) data and Hysplit backward trajectories on days with peak concentrations of retene: **a** Fire spots detected from April 21 st to 28th 2020. **b** Backward air masses trajectories on April 25th 2020 and **c** April 28th 2020. **d** Fire spots detected from July 22nd to 26th 2020. **e** Backward air masses trajectories on July 26th 2020

Retene, a marker of biomass burning and mutagenic PAH (Peixoto et al. 2019; Ramdhal 1983), showed no change in median concentration compared to a previous study carried out from winter to summer at the same sampling site (0.102 ng m^−3^, Serafeim et al. 2023). It suggests that the contribution of biomass burning did not change during or after the pandemic. The presence of retene in PM_2.5_ in São Paulo is influenced by the transport of air masses. In the present study, backward air masses’ trajectories were analyzed, and fires (INPE 2020, 2022) were observed 3 days before the sampling day with the highest retene levels (Fig. 5 and 6). These trajectories indicated the influence of plumes originating from various regions experiencing biomass burning, including areas of savanna (Cerrado), Atlantic Forest, and agro-industrial zones.

**Fig. 7:**
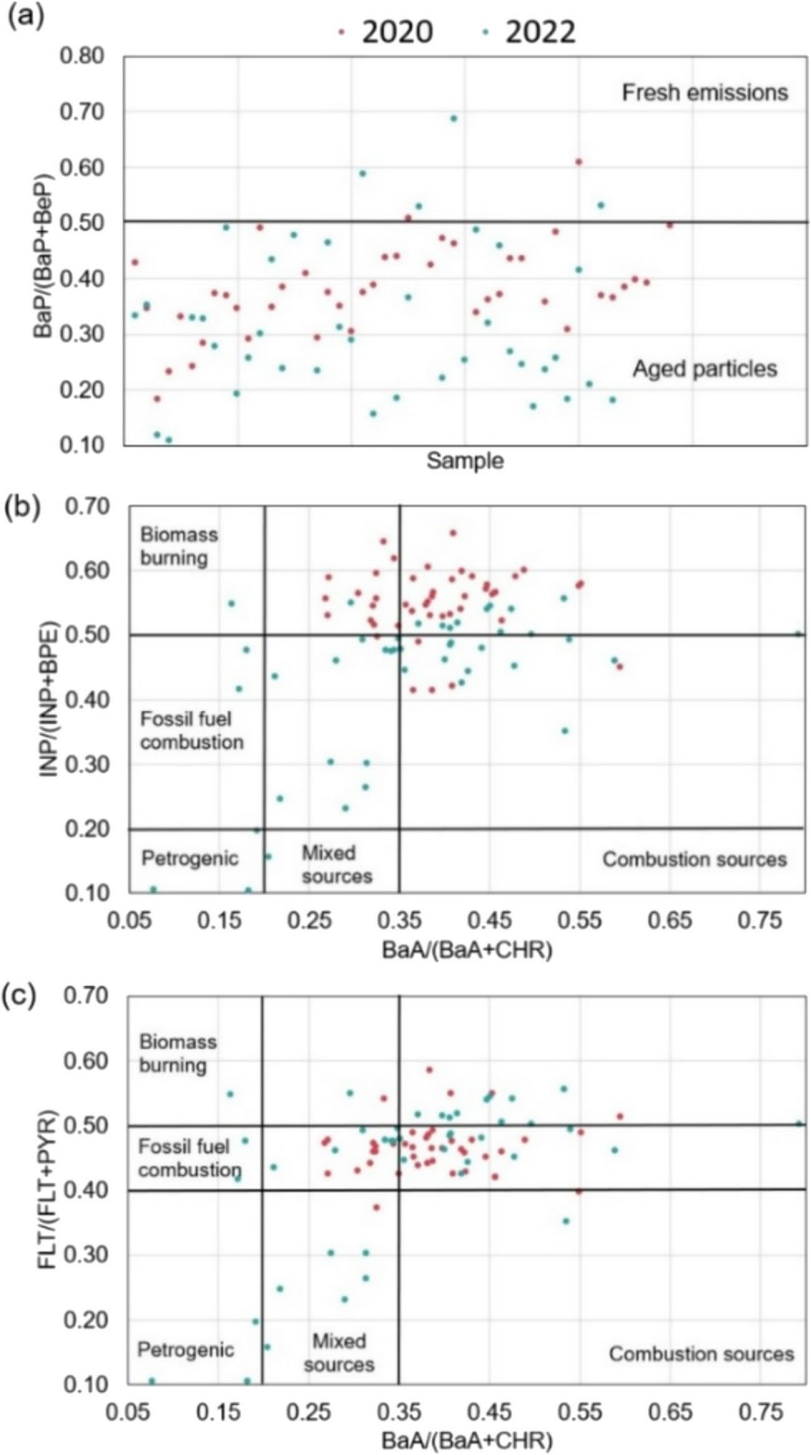
PAHs diagnostic ratios: **a** BaP/(BaP+BeP), **b** INP/(INP+BPE) vs. BaA/(BaA+CHR), and **c** FLT/(FLT+PYR) vs BaA/(BaA+CHR)

Diagnostic ratios of PAHs were used to estimate PM_2.5_ aging and its emission sources (Fig. 7). The BaP/(BeP+BaP) ratio is presented in Fig. 7a. Combustion sources emit equal amounts of BaP and BeP isomers. However, BaP is more susceptible to photooxidative processes, so values closer to 0.5 indicate recent emission. In this case, the values found indicated non-recent emission and the aging of the PM_2.5_ through photooxidation in both years. These aged particles can also be associated with the transport of pollutants from other regions.

BaA/(BaA+CHR) ratios are presented in Fig. 7b and c. Most sampling days had ratios above 0.35, suggesting that combustion processes were the main sources in both campaigns (Zhang et al. 2008; Yunker et al. 2002). The INP/(INP+BPE) ratios (Fig. 7b) indicated that biomass burning was the main source of these compounds during the pandemic, while fossil combustion became more significant after the economic reopening. Conversely, the FLT/(FLT+PYR) ratios (Fig. 7c) reflected the impact of fossil fuel combustion in 2020 and the dominance of biomass burning in 2022. The potential influence of photochemical reactions on the diagnostic ratio should not be ignored. FLT is known to be more photostable than its isomer, which can cause the FLT/(FLT+PYR) ratio to increase during photochemical aging (Ray et al. 2019). Additionally, BPE has a shorter half-life than INP, which can affect the INP/(INP+BPE) ratio (Niu et al. 2007; Tobiszewski and Namieśnik 2012).

In São Paulo, vehicular traffic is the primary source of pollutants, but biomass burning contributions are always evident. Although residential biomass burning is uncommon in the city, the transport of pollutants from regions with forest fires and agro-industrial burning also contributes to the PAH concentrations. Additionally, restaurants and pizzerias that use wood-fired ovens serve as local sources. Despite restrictions on in-person activities and the closure of these establishments during the pandemic, delivery services continued and even expanded. The findings of the present study showed that, unlike other anthropogenic sources, biomass burning was not affected by the restriction measures.

In the source apportionment by factor analysis, the sum of PAHs was associated primarily with biomass burning, with minor contributions from vehicular emissions. However, the high abundance of PAHs associated with vehicular traffic (e.g., BbF, BPE, and COR) and the diagnostic ratios indicated that both sources had significant contributions. PAHs showed strong correlations (*ρ* > 0.70) with carbonaceous species and biomass burning markers (anhydride monosaccharides and Rb), and the variability of this source may have been dominant in the factor analysis.

### Oxy and nitro-PAHs

As observed for PAHs and other pollutants in PM_2.5_, the oxy-PAHs showed higher concentrations in 2022 (*p* < *0.05*) than in 2020. No significant differences were found for nitro-PAHs, but these compounds had lower detection frequencies during the lockdown (2–22%) compared to the same period of 2022 (27–64%).

The concentrations of oxy and nitro-PAHs in 2020 were similar to or even higher than those reported in a study conducted during the pre-pandemic period, from June to December 2019, when the median concentrations of total oxy-PAHs and nitro-PAHs were 366 pg m^−3^ and 90.4 pg m^−3^, respectively (Serafeim et al. 2023). This study covered winter, spring, and summer, including a period with frequent rainfall, which may have contributed to the reduction of these pollutants in the atmosphere (Serafeim et al. 2023).

9,10-AQ was the most abundant oxy-PAH in both years, followed by 7,12-BaAQ in 2020 and 2-MAQ in 2022. Secondary formation and primary diesel emissions are sources of oxy-PAHs in the air. Photochemical reactions of anthracene and primary diesel emissions are identified as the main sources of 9,10-AQ (Nocun and Schantz 2013; Mmereki et al. 2004). 7,12-BaAQ can be emitted by diesel combustion or formed through secondary reactions (Albinet et al. 2007; Zhao et al. 2021), and 2-MAQ has already been linked to vehicular emissions (Alam et al. 2013).

Among the nitro-PAHs, 2+3-NFLT and 1-NPYR were the most abundant during the lockdown period. After economic reopening, 9-NANT was the most prevalent, followed by 2+3-NFLT. These compounds can also originate from primary emissions or secondary processes. 1-NPYR is linked to primary diesel emissions, while 2+3-NFLT is mainly associated with secondary formations (Zhao et al. 2021). A 2+3-FLT/1-NPYR ratio lower than 5 indicates the dominance of direct emissions (Tomaz et al. 2017). In 2020, this ratio was below 5 in all samples where both compounds were detected (1.4–2.0), indicating primary emissions as the main source during those days. In 2022, the 2+3-FLT/1-NPYR varied from 1 to 10, showing contributions from both secondary formation and primary emissions.

The increase in the atmosphere’s oxidative capacity and secondary organic formation, due to higher ozone levels, was observed at most urban sites during the lockdown period (Sun et al. 2020; Xu et al. 2022). However, Xu and collaborators (2022) reported a decrease in nitro and oxy-PAHs in this period, which were linked to reduced emissions of PAH precursors. Similarly, in the present study, the secondary formation of nitro-PAHs during the lockdown period may also have been limited by the availability of precursors.

Nitro-PAHs are usually found in concentrations 1 to 3 orders of magnitude lower than their PAH precursors and are subject to photooxidation and reactions with radicals (OH and NO_3_) and gases (O_3_, NO_2_, N_2_O_5_) in the atmosphere (Bandowe and Meusel 2017). Despite the decreased concentrations, these compounds still pose a risk to human health. Oxy and nitro-HPAs are linked to oxidative stress and DNA adduct formation, and due to their direct mutagenic effect, these compounds are more carcinogenic than their precursor PAHs (Idowu et al. 2019).

### Health risk assessment due to PAHs and metals exposure

To evaluate the risk of exposure to PM_2.5_ to human health, toxicity indices based on pollutant concentrations were calculated as described in the literature (Yassaa et al. 2001). The Benzo(a)pyrene-equivalent index (BaPE) was determined to estimate the environmental exposure risk to PAHs for human health, considering PAH concentrations found in samples and the carcinogenic potential of each compound relative to BaP (Yassaa et al. 2001).

As expected, the BaPE index values were higher in 2022 (0.1–9.3 ng m^−3^) than during the pandemic (0.01–1.90 ng m^−3^). As a consequence of the reduction in emissions during the lockdown period, the BaPE index in 2020 was also lower than those reported previously in São Paulo (Table 5), including those found between June and December 2019 (0.1–2.3 ng m^−3^), the period comprising the wet season (Serafeim et al. 2023). The limit of 1 ng m^−3^ recommended by the World Health Organization (WHO 2021b) was exceeded in 4 days of sampling in 2020 and 23 days in 2022.

**Table 5:** Benzo(a)pyrene equivalent (BaPE) index in São Paulo PM_2.5_

	BaPE (ng m^−3^)			Period
	Min–max	Average	Median	
Present study	0.01–1.9	0.50	0.32	March to August 2020 (lockdown)
Present study	0.1–9.3	1.81	1.15	March to August 2022 (after economic reopening)
Serafeim et al. (2023)	0.1–2.3	--	0.50	June to December 2019
Alves et al. (2020)	0.2–6.1	1.3	--	August 2015 to February 2016

The BaP-TEQ and BaP-MEQ indices indicate that the mutagenic effects of PAHs had a greater impact on cancer risk in 2020 (Table 6). The median values for BaP-TEQ and BaP-MEQ during the social restriction period were 0.42 and 0.8 ng m^−3^, respectively. In that year, BaP-MEQ exceeded 1 ng m^−3^ for 14 days, and BaP-TEQ did so for 8 days. After the economic reopening, a larger increase in carcinogenic effects was observed. In 2022, the median BaP-TEQ and BaP-MEQ were 3.8 and 1.7 ng m^−3^, respectively, and these indices surpassed the safe limit on 40 and 28 days.

**Table 6:** BaP-TEQ, BaP-MEQ, and ILCR_inh_ for children and adults in 2020 and 2022

Year	Index	Min–max	Median	Average
2020	BaP-TEQ (ng m^−3^)	0.014–4.7	0.42	0.74
	BaP-MEQ (ng m^−3^)	0.036–3.5	0.80	0.97
	ILCRinh children (10 years)	4.3×10^–9^–1.4×10^–6^	1×10^–7^	2×10^–7^
	ILCRinh adults (70 years)	1.1×10^–8^–3.6×10^–6^	3×10^–7^	6×10^–7^
2022	BaP-TEQ (ng m^−3^)	0.4–28.2	3.8	6.4
	BaP-MEQ (ng m^−3^)	0.2–12.0	1.7	2.6
	ILCRinh children (10 years)	1×10^–7^–8×10^–6^	1.1×10^–6^	2×10^–6^
	ILCRinh adults (70 years)	3×10^–7^–2×10^–5^	3×10^–6^	5×10^–6^

The incremental lifetime cancer risk due to inhalation exposure to PAHs (ILCR_inh_) was calculated (Table 6). According to the EPA, ILCR below 1 × 10^–6^ indicates a negligible lifetime cancer risk, ILCR between 1 × 10^–6^ and 1 × 10^–4^ indicates moderate cancer risk, and ILCR above 10^–4^ indicates a high cancer risk (U. S. Epa 2004, 2009; Famiyeh et al. 2021). During the pandemic period, the ILCR_inh_ values remained below 1 × 10^–6^ on most sampling days, but exceeded this threshold in 2 days for children and 7 days for adults. The median ILCR_inh_ during the lockdown was 1.2 × 10^–7^ for children, and 3.2 × 10^–7^ for adults. The values found in this work are lower than those reported by Feng and collaborators (2023) in Shanghai (China) and by Ambade and collaborators (2021) in Jamshedpur (India) during the period of restrictive measures. In these studies, ILCR values surpassed 1 × 10^–6^ threshold but stayed below 1 × 10^–4^.

In 2022, the cancer risk increased and exceeded the safe limit (1 × 10^–6^) in 23 days for children and 39 days for adults, but it remained below the high cancer risk (1 × 10^–4^). That year, the median ILCR_inh_ was 1.1 × 10^–6^ for children and 2.9 × 10^–6^ for adults. In a megacity with over 12 million inhabitants, these results translate to 12 additional cancer cases in children and 36 in adults, based on inhalation exposure to carcinogenic PAHs. Although these values are still lower than those found in other megacities in developing countries, where these often exceed the 1 × 10^–4^ limit (Yu et al. 2020; Singh et al. 2023).

Non-carcinogenic and carcinogenic risks from metal exposure were also assessed (Table 7). The safe limit for the non-carcinogenic risk, indicated by the hazard quotient (HQ), is reported as 1 (Alves et al. 2020). A study conducted in São Paulo between the winter of 2015 and summer 2016 found a calculated HQ sum of 1.1, exceeding the safe level (Alves et al. 2020). In this study, the individual HQ values calculated for all metals and the sum of HQ were below 1 for both during and after the pandemic periods. During the lockdown period, the individual CR values for children did not exceed the safety threshold established by the EPA (CR = 1 × 10^–6^, Alves et al. 2020; U.S. Epa 2001), but the total CR values (median 1.3 × 10^–6^) indicated cancer risk on most sampling days. For adults, the individual CR of Cr (median 4.1 × 10^–6^) and the total CR values (median 5.2 × 10^–6^) surpassed the safe limit.

**Table 7:** Non-carcinogenic risk (HQ) and carcinogenic risk (CR) due to inhalation exposure to trace elements in São Paulo in 2020 and 2022

	2020			2022		
	HQ	CR_children_	CR_adult_	HQ	CR_children_	CR_adult_
Cr	0.014	3.3 × 10^–7^	1.3 × 10^–6^	0.010	7.0 × 10^–6^	2.8 × 10^–5^
Mn	0.052	--	--	0.129	--	--
Ni	0.044	9.0 × 10^–9^	3.6 × 10^–8^	0.008	1.6 × 10^–8^	6.3 × 10^–8^
As	0.023	1.2 × 10^–7^	4.7 × 10^–7^	0.034	1.9 × 10^–7^	7.4 × 10^–7^
Cd	0.026	3.6 × 10^–8^	1.4 × 10^–7^	0.027	4.2 × 10^–8^	1.7 × 10^–7^
Pb	–	4.2 × 10^–8^	1.7 × 10^–7^	--	5.0 × 10^–8^	2.0 × 10^–7^
Sum	**0.16**	**5.3 × 10**^**–7**^	**2.1 × 10**^**–9**^	**0.19**	**7.2 × 10**^**–6**^	**2.9 × 10**^**–5**^

In 2022, the cancer risk threshold was surpassed for individual Cr exposure for children (median 7.0 × 10^–6^) and adults (median 2.8 × 10^–5^). The median CR was 7.2 × 10^–6^ for children and 2.8 × 10^–5^ for adults. These values are higher than those reported in a previous study conducted in São Paulo, where the sum of CR_children_ and CR_adults_ was 1.4 × 10^–6^ and 5.7 × 10^–6^, respectively (Alves et al. 2020).

The risks due to element exposure found in this study were lower than those reported in Chinese cities before and during the COVID-19 pandemic. In a study conducted in Shanghai, the sum and individual CR values were below the threshold limit for children, but the CR for As and the total CR were above the limits for adults during the lockdown period (Wang et al. 2022). Yet, a decrease in carcinogenic risks compared to the pre-lockdown period was observed due to a reduction in metal emissions. CR and HQ values above safe limits were also reported in Linfen (China), where the individual and sum of CR were above 10^–6^ for adults and children during the lockdown, and above 10^–4^ during the pre-lockdown and partial lockdown periods. The non-carcinogenic risk (HQ) also exceeded the recommended limit for children and adults but showed a decline during the social isolation (Wang et al. 2021b).

## Conclusions

PM_2.5_ presented significant variation during and after the COVID-19 pandemic. As expected, the concentrations decreased during the lockdown period (2020) compared to the previous year and increased after the economic reopening (2022). The variation in anthropogenic activities drove the changes in air pollution.

The source apportionment by the FA-MLR approach showed contributions of vehicular traffic, biomass burning, and secondary formation. The primary sources were biomass burning in 2020 and vehicular traffic in 2022. It is noteworthy that biomass burning was an important contributor to PM_2.5_ concentrations at this site, and its contribution did not change with the economic reopening.

Changes in vehicular fuel consumption and pollutant emissions were observed between the pre-pandemic and post-pandemic periods. Organic (PAHs and derivatives) and inorganic (WSIs and elements) pollutants linked to fossil fuel combustion, vehicular traffic, and dust resuspension increased significantly with the economic reopening in 2022. Contrary to expectations, Cu and Mo, metals linked to ethanol-fueled vehicles’ emissions, decreased in 2022. These results were related to the increase in the price of this fuel and its decreased competitiveness compared to gasoline.

The carbonaceous species indicated that biomass burning was a significant source of pollutants. No notable differences were observed in the concentrations of species associated with biomass burning, suggesting there was no decrease in this activity during the pandemic. The variation in L/M and L/G ratios suggested the burning of different biomass types in both years, and that local (e.g., pizzerias, bakeries) and outside sources (forest fires and agricultural burning) contributed to the observed values. On days with high concentrations of biomass-burning markers (retene and anhydride monosaccharides), air masses passing over burning areas in other states and in the interior of São Paulo showed that forest fires and agricultural burning contributed to the air pollution.

The analysis of health risk indices showed an increase in carcinogenic potential in the samples collected after the pandemic; in addition, significant cancer risk due to PAH inhalation was observed in most samples. The non-carcinogenic risk indices (HQ) due to exposure to toxic metals remained below the safe limit, but the carcinogenic indices (CR) showed higher cancer risks after the economic reopening.

## Supplementary information

Below is the link to the electronic supplementary material.

ESM 1 (DOCX 1.19 MB)

ESM 2 (XLSX 171 KB)

## Data Availability

All data supporting the findings of this paper are presented in the manuscript and supplementary information. If necessary, data can be requested from the corresponding author.
