# Supplementary material for: Changes in air composition driven by differences in biofuel consumption
Source: Environ Sci Pollut Res Int. 2026 Apr 20;33(15):7017–41. doi: 10.1007/s11356-026-37733-9 (PMC13156166; doi:10.1007/s11356-026-37733-9)
Supplement: Supplementary file 1 — (DOCX 1.19 MB) [file 11356_2026_37733_MOESM1_ESM.docx]

**Changes in air composition driven by differences in biofuel consumption**

Camila Novais Farias^a*^, Débora Pagliuso^b^, Adriana Grandis^b^, José Vinícius Martins^c^, Clara Rodrigues Pereira^d^, Lilian Lefol Guarieiro^d^, Paulo Eduardo Artaxo Netto^e^, Marcos Silveira Buckeridge^b^, Pérola de Castro Vasconcellos^a^

^a^ Department of Chemistry, Institute of Chemistry, University of São Paulo, 05508-000, São Paulo, Brazil

^b^Department of Botany, Institute of Biosciences, University of São Paulo, 05508-090, São Paulo, Brazil

^c^Department of Mineralogy and Geotectonics, Institute of Geosciences, University of São Paulo, 05508-080, São Paulo, Brazil

^d^SENAI CIMATEC University, 41650-010, Salvador, Bahia, Brazil

^e^Department of Applied Physics, Institute of Physics, University of São Paulo, 05508-090, São Paulo, Brazil

^*^ Corresponding authors:

Camila Novais Farias, e-mail: [camila.farias@usp.br](mailto:camila.farias@usp.br), [camila.farias@alumni.usp.br](mailto:camila.farias@alumni.usp.br), phone: +55 (11) 3091-9109

**Contents**

1. **Sampling site**

Fig. S1. The sampling site in São Paulo (prepared using Google Earth, coordinates: 23° 33’ 56.57’’ S; 46° 43’ 35.16’’ W).

Table S1. Meteorological conditions in São Paulo in 2020 and 2022.

Equation S1. Non-sea-salt potassium calculation.

1. **Analytical data**

**2.1 Additional text: Relative standard deviation, limits of detection, and quantification**

Table S2. Analytical information on carbonaceous and inorganic species (OC, EC, WSIs, and elements).

Table S3. Analytical information on monosaccharides.

Table S4. Analytical information on polycyclic aromatic hydrocarbons (PAHs) and derivatives (Oxy and nitro-PAHs).

1. **Health Risk Assessment due to metal exposure**

Table S5. Reference concentrations (RfC) and Unit Risk (IUR) for cancer risk (ECR) and non-carcinogenic-risk (HQ) assessment (USEPA, 2023).

1. **Statistical analysis results**

Table S6. Shapiro-Wilk and Mann-Whitney tests results. (attached in Excel, Online Resource 2)

Table S7. Spearman correlation – data 2020. (attached in Excel, Online Resource 2)

Table S8. Spearman correlation – data 2022. (attached in Excel, Online Resource 2)

1. **Source apportionment: factor analysis – multiple linear regression (FA-MLR)**

**5.1 Factor Analysis (FA)**

Table S9. Bartlett’s and Kaiser-Meyer-Olkin (KMO) tests.

Fig. S2. Scree plot graphs: (a) 2020 and (b) 2022.

Table S10. Summary of factor analysis results.

Fig. S3. Cook's distance graph - 2022.

**5.2 Multiple Linear Regression (MLR)**

Table S11. Summary of multiple linear regression (MLR) results.

Table S12. Coefficients of multiple linear analysis (MLR) – 2020.

Table S13. Coefficients of multiple linear analysis (MLR) – 2022.

Fig. S4. Reconstruction of PM_2.5_ concentrations by FA-MLR in 2020: Calculated (Yellow) vs. Observed (Pink).

Fig. S5. Reconstruction of PM_2.5_ concentrations by FA-MLR in 2022: Calculated (Yellow) vs. Observed (Blue), and Outlier removed (sample 41, Red).

- 1. **Additional text – Fundamentals of FA-MLR analysis and calculation of source contributions**

1. **Sampling site**


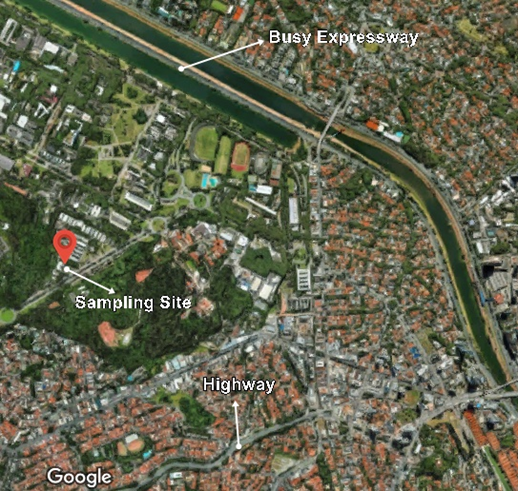


**Fig. S1.** The sampling site in São Paulo (prepared using Google Earth, coordinates: 23° 33’ 56.57’’ S; 46° 43’ 35.16’’ W).

**Table S1.** Meteorological data in São Paulo in 2020 and 2022 (IAG, 2022).

| **Meteorological variable** | **2020** | | **2022** | |
| --- | --- | --- | --- | --- |
|  | **Range** | **Average** | **Range** | **Average** |
| Average temperature (ºC) | 13-22 | 18 | 10-25 | 18 |
| Average relative humidity (%) | 51-95 | 77 | 56-95 | 76 |
| Solar radiation (MJ m^-2^) | 3.9-24.3 | 15.3 | 3.7-20.6 | 15.2 |
| Atmospheric pressure (hPa) | 923-936 | 928 | 922-934 | 927 |
| Precipitation (mm) | 0-18.0 | 2.6* | 0-6.2 | 2.3* |
| Average wind speed (km h^-1^) | 2.1-12.8 | 5.2 | 1.8-11.0 | 5.6 |
| Precipitation frequency (%) | 37 | | 16 | |

^*Only days with rainfall occurrence were considered in the average precipitation.^

The non-sea-salt potassium (nss-K^+^) is estimated based on the mass ratio of potassium to sodium in seawater (0.036) (George et al. 2008), as described below:

$nss{-K}^{+} = C_{K+} - 0.036 \times C_{Na+}$ (Eq. S1)

Where C_K+_ is the concentration of potassium and C_Na+_ is the concentration of sodium in PM_2.5_ samples.

1. **Analytical data**

**2.1 Additional text: Relative standard deviation, limits of detection, and quantification**

The errors associated to the measures are represented by the relative standard deviation (RSD), expressed as a percentage and calculated as follows:

$RSD \left( \% \right) = \frac{sd}{\bar{x}}\times100\%$ (Eq. S4)

where *sd* is the standard deviation of replicates and $\bar{x}$ is the average of the measures.

The limits of detection (DL) and quantification (QL) of carbonaceous species determined by termooptical analysis were provided by the manufacturer (Brown et al., 2020). For other species, DL and QL were determined according to the methods described by Shrivastava and Gupta (2011). DL and QL for water-soluble ions (WSIs), monosaccharides, polycyclic aromatic hydrocarbons (PAHs), and their derivatives (oxy and nitro-PAHs) were determined following a visual method. Successive dilutions of standard solutions were analyzed, and the DL was defined as the concentration at which the signal of the analyte corresponded to 3 times the noise level. The quantification limit was calculated by multiplying the DL by three (QL = 3 × DL). For elements, DL and QL were calculated from the parameters of the analytical curve, as described in Equations S3:

$$Limit=F\times\frac{sd}{b} (Eq. S2)$$

where *F* is a factor of 3.3 for DL and 10 for QL, *sd* is the standard deviation of the intercept, and *b* is the intercept of the analytical curve.

**Table S2**. Analytical information on carbonaceous and inorganic species (OC, EC, WSIs, and elements).

| **Species** | **DL (µg C cm^-2^)** | **DQ (µg C cm^-2^)** | **RSD (%)** |
| --- | --- | --- | --- |
| **OC** | 0.4 | 1.2 | 2.0 |
| **EC** | 0.2 | 0.6 | 0.2 |
| **WSI** | **(ng mL^-1^)** | **(ng mL^-1^)** |  |
| Fluoride | 10 | 40 | 7 |
| Formate | 10 | 30 | 5 |
| Chloride | 30 | 80 | 9 |
| Nitrate | 10 | 30 | 7 |
| Sulfate | 10 | 40 | 5 |
| Oxalate | 10 | 40 | 7 |
| Sodium | 20 | 70 | 2 |
| Ammonium | 30 | 100 | 8 |
| Potassium | 20 | 60 | 1 |
| Calcium | 40 | 120 | 7 |
| Magnesium | 50 | 150 | 6 |
| **Elements** | **(ng g^-1^)** | **(ng g^-1^)** |  |
| Ti | 0.026 | 0.078 | 2.0 |
| V | 0.012 | 0.037 | 2.7 |
| Cr | 0.034 | 0.102 | 1.1 |
| Mn | 0.034 | 0.102 | 1.8 |
| Fe | 1.670 | 5.010 | 2.2 |
| Ni | 0.010 | 0.031 | 6.7 |
| Cu | 0.016 | 0.048 | 1.2 |
| Zn | 0.630 | 1.890 | 1.5 |
| As | 0.021 | 0.063 | 3.4 |
| Se | 0.00 | 0.00 | 5.4 |
| Rb | 0.027 | 0.081 | 1.8 |
| Sr | 0.004 | 0.012 | 1.7 |
| Mo | 0.006 | 0.019 | 1.0 |
| Cd | 0.023 | 0.069 | 1.4 |
| Sn | 0.007 | 0.020 | 1.6 |
| Sb | 0.010 | 0.031 | 2.0 |
| Ba | 0.048 | 0.144 | 1.2 |
| Pb | 0.009 | 0.026 | 1.2 |

**Table S3.** Analytical information on monosaccharides.

| Compound | **DL (ng mL^-1^)** | **DQ (ng mL^-1^)** | **RSD (%)** |
| --- | --- | --- | --- |
| Levoglucosan | 0.3 | 0.9 | 7.2 |
| Mannosan | 0.6 | 1.8 | 4.2 |
| Galactosan | 0.3 | 0.9 | 3.3 |
| Glucose | 0.3 | 0.9 | 1.5 |
| Mannose | 0.3 | 0.9 | 1.5 |
| Galactose | 0.3 | 0.9 | 1.7 |
| Xylose | 0.3 | 0.9 | 2.0 |
| Fucose | 0.3 | 0.9 | 2.1 |
| Arabinose | 0.3 | 0.9 | 2.0 |
| Rhamnose | 0.3 | 0.9 | 2.4 |

**Table S4.** Analytical information on polycyclic aromatic hydrocarbons (PAHs) and derivatives (Oxy and nitro-PAHs).

| **Compound** | **DL (ng mL^-1^)** | **DQ (ng mL^-1^)** | **Recovery (%)** | **RSD (%)** |
| --- | --- | --- | --- | --- |
| **PAH and derivatives** |  |  |  |  |
| Phenanthrene | 2.5 | 7.5 | 123 | 8 |
| Fluroanthene | 1.25 | 3.75 | 109 | 8 |
| Pyrene | 1.25 | 3.75 | 129 | 10 |
| Retene | 5 | 15 | 130 | 7 |
| Benzo(a)anthracene | 5 | 15 | 127 | 6 |
| Chrisene | 5 | 15 | 92 | 5 |
| Benzo(b)fluoranthene | 10 | 30 | 90 | 4 |
| Benzo(k)fluoranthene | 7.5 | 22.5 | 87 | 5 |
| Benzo(a)pyrene | 7.5 | 22.5 | 102 | 5 |
| Benzo(e)pyrene | 10 | 30 | 87 | 8 |
| Perylene | 10 | 30 | 102 | 5 |
| Indeno(1,2,3-c,d)pyrene | 10 | 30 | 87 | 4 |
| Dibenzo(a,h)anthracene | 10 | 30 | 126 | 3 |
| Benzo(g,h,i)perylene | 10 | 30 | 121 | 10 |
| Coronene | 10 | 30 | 116 | 2 |
| 9-Fluorenone | 5 | 15 | 93 | 8 |
| 9,10-Anthraquinone | 5 | 15 | 98 | 5 |
| 2-methylathraquinone | 10 | 30 | 97 | 6 |
| Benzoanthraquinone | 20 | 60 | 104 | 10 |
| 9-nitroanthracene | 25 | 75 | 93 | 4 |
| 1-nitropyrene | 25 | 75 | 94 | 3 |
| 3-nitrofluoranthene | 25 | 75 | 88 | 9 |
| 6-nitrochrisene | 50 | 150 | 102 | 8 |

1. **Health Risk Assessment due to metal exposure**

**Table S5.** Reference concentrations (RfC) and Unit Risk (IUR) for cancer risk (ECR) and non-carcinogenic risk (HQ) assessment (U.S. EPA, 2023).

| Specie | IUR (µg m^-3^)^-1^ | RfC (mg m^-3^) |
| --- | --- | --- |
| Cr (IV) | 1.1 ∙ 10^-2^ | 3.0 ∙ 10^-5^ |
| Mn | -- | 5.0 ∙ 10^-5^ |
| Ni (soluble salt) | 2.6 ∙ 10^-4^ | 1.0 ∙ 10^-5^ |
| As | 4.3 ∙ 10^-3^ | 1.5 ∙ 10^-5^ |
| Cd (diet) | 1.8 ∙ 10^-3^ | 1.0 ∙ 10^-5^ |
| Pb (acetate) | 8.0 ∙ 10^-5^ | -- |

1. **Spearman’s correlations of chemical species of PM_2.5_**

Table S6. Shapiro-Wilk and Mann-Whitney test results. (attached in Excel, Online resource 2)

Table S7. Spearman correlation – data 2020. (attached in Excel, Online resource 2)

Table S8. Spearman correlation – data 2022. (attached in Excel, Online resource 2)

1. **Source apportionment: factor analysis – multiple linear regression (FA-MLR) results**
   1. **Factor analysis (FA)**

**Table S9.** Bartlett’s and Kaiser-Meyer-Olkin (KMO) tests.

|  | **Parameter** | **2020** | **2022** |
| --- | --- | --- | --- |
| **Bartlett’s test** | χ^2^ | 968.5338 | 1054.32 |
|  | p-value | 1.062365e^-97^ | 1.666415e^-112^ |
|  | df | 210 | 210 |
| **KMO factor adequacy** | Overall MSA | 0.71 | 0.75 |

The KMO and Bartlett’s tests were conducted to assess the adequacy of the sampling for factor analysis (FA).

**Bartlett’s test of sphericity:**

The chi-square (χ^2^) statistic indicates whether the correlation matrix is different from the identity matrix.

A significant chi-square (*p-value<0.05*) indicates a good adequacy of the data for factor analysis.

df = degrees of freedom of the correlation matrix.

**Kaiser-Meyer-Olkin (KMO) test:**

An overall MSA (measure of sampling adequacy) > 0.70 in the KMO test indicates the suitability of the data for factor analysis.

**
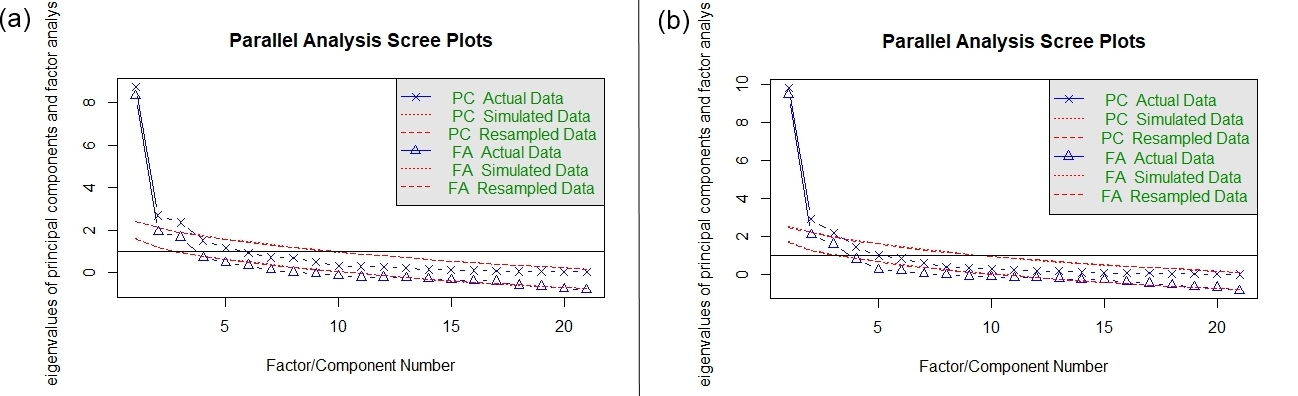
**

**Fig. S2.** Scree plot graphs: (a) 2020 and (b) 2022.

**Table S10.** Summary of factor analysis results.

|  | **2020** | | | **2022** | | |
| --- | --- | --- | --- | --- | --- | --- |
|  | **F1** | **F2** | **F3** | **F1** | **F2** | **F3** |
| **Proportion var** | 0.315 | 0.160 | 0.133 | 0.356 | 0.176 | 0.143 |
| **Cumulative var** | 0.315 | 0.475 | 0.608 | 0.356 | 0.532 | 0.676 |

- 1. **Multiple linear regression (MLR)**

**
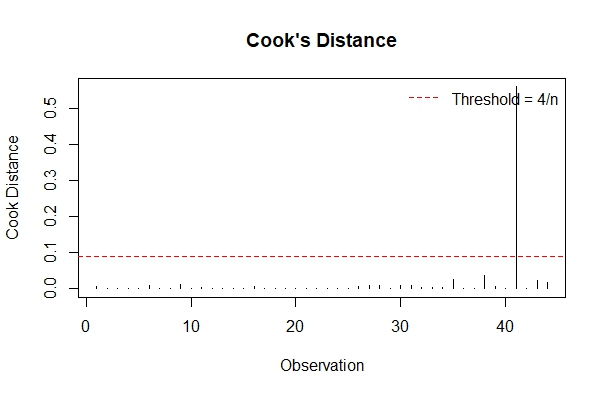
**

**Fig. S3.** Cook's distance graph - 2022. Outlier removed: sample 41 (August 21^st^, 2022).

**Table S11.** Summary of multiple linear regression (MLR) results.

| **Parameter** | **2020** | **2022 (without outlier)** |
| --- | --- | --- |
| **Residual standard error** | 2.585 on 45 degrees of freedom | 4.195 on 39 degrees of freedom |
| **Multiple R-squared** | 0.898 | 0.8793 |
| **Adjusted R-squared** | 0.8912 | 0.87 |
| **F-statistic** | 132 on 3 and 45 DF | 94.69 on 3 and 39 |
| **p-Value** | < 2.2e-16 | < 2.2e-16 |

**Table S12.** Coefficients of multiple linear analysis (MLR) – 2020.

| **Cofficients** | **Estimate** | **Std. Error** | **T value** | **p-value** |
| --- | --- | --- | --- | --- |
| Intercept | 3.9756 | 0.8358 | 4.756 | < 2.06e-05 *** |
| F1 | 6.0640 | 0.3727 | 16.271 | < 2e-16 *** |
| F2 | 2.9768 | 0.3864 | 7.703 | 9.35e-10 *** |
| F3 | 3.2552 | 0.3856 | 8.442 | 7.90e-11 *** |

^Signif. codes: 0 ‘***’ 0.001 ‘**’ 0.01 ‘*’ 0.05 ‘.’ 0.1 ‘ ’ 1^

**Table S13.** Coefficients of multiple linear analysis (MLR) – 2022.

| **Cofficients** | **Estimate** | **Std. Error** | **T value** | **p-value** |
| --- | --- | --- | --- | --- |
| Intercept | 2.3884 | 1.7063 | 1.400 | 0.17 |
| F1 | 8.3639 | 0.6464 | 12.939 | 1.08e-15 *** |
| F2 | 5.5289 | 0.6339 | 8.722 | 1.06e-10 *** |
| F3 | 4.3367 | 0.6377 | 6.801 | 4.03e-08 *** |

^Signif. codes: 0 ‘***’ 0.001 ‘**’ 0.01 ‘*’ 0.05 ‘.’ 0.1 ‘ ’ 1^

**
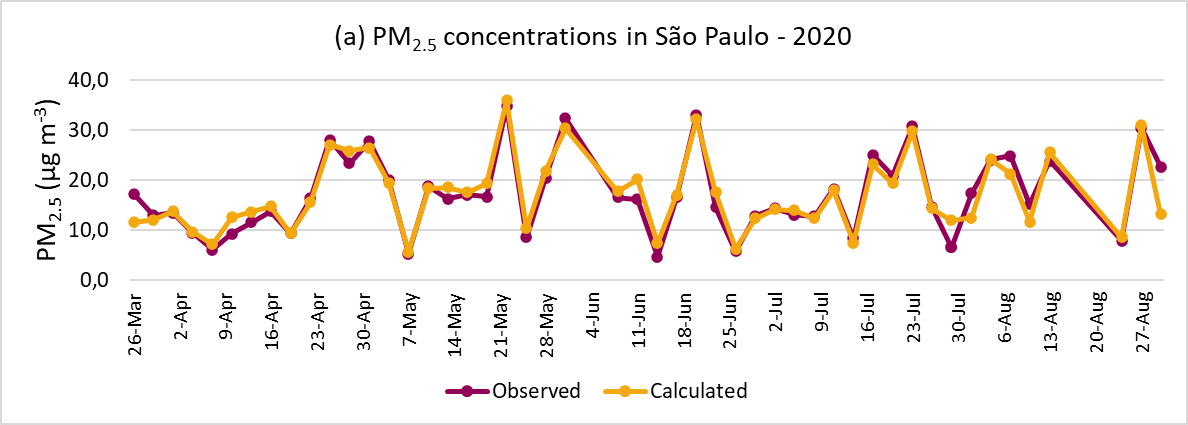
**

**Fig. S4.** Reconstruction of PM_2.5_ concentrations by FA-MLR in 2020: Calculated (Yellow) vs. Observed (Pink).

**
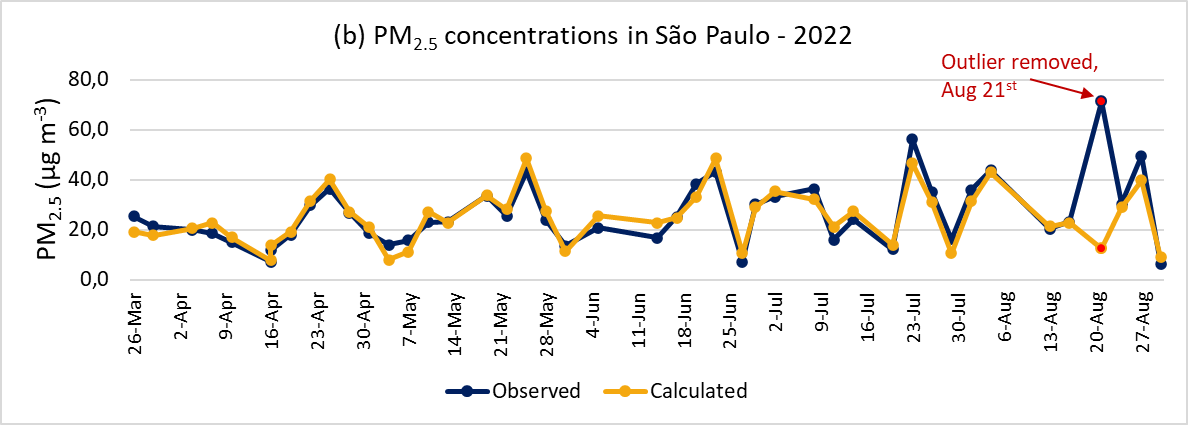
**

**Fig. S5.** Reconstruction of PM_2.5_ concentrations by FA-MLR in 2022: Calculated (Yellow) vs. Observed (Blue), and Outlier removed (sample 41, Red).

- 1. **Additional text – Fundamentals of FA-MLR analysis and calculation of source contributions**

The factor analysis (FA) was performed to reduce data variability, as described in Section 2.11 (main text). The profiles of the sources were described by the loading graphs (Fig. 1, Section 3.2).

The first step of FA includes the standardization (autoscaling) of the raw data as follows:

$Z_{ik}=\frac{C_{ik}-\bar{C_{i}}}{\sigma_{i}}$ (Eq. S4)

Where *i* is the number of variables in the analysis, *k* is the number of observations (samples), $\bar{C}_{i}$ is the average concentration of variable *i* over all sampling data, $C_{ik}$ is the concentration of variable *i* for each observation, $\sigma_{i}$ is the standard deviation of parameter *i*, and Z_ik_ (Z-score) represents the concentrations after the standardization.

The quantitative determination of the contributions of the factors to PM_2.5_ mass requires the use of raw-unit scores (absolute scores). Therefore, the factor scores were unnormalized following the methodology described by Thurston and Spengler (1985).

The calculation of the absolute factor scores (AFS) involves the introduction of an artificial zero-concentration sample:

${(Z_{0})}_{i}=\frac{0-\bar{C_{i}}}{\sigma_{i}}=\frac{-\bar{C_{i}}}{\sigma_{i}}$ (Eq. S5)

Then, the scores of the zero-sample were calculated similarly to the sample scores. In the *psych* package, the factor scores are calculated by multiplying the data matrix by the weight matrix (Revelle et al., 2025), as described in the equations S6 and S7:

${Fs}_{jk} = \sum_{i=1}^{m} (W_{ij}\times Z_{ik})$ (Eq. S6)

${Fs}_{j0} = \sum_{i=1}^{m} (W_{ij}\times Z_{i0})$ (Eq. S7)

Where FS_jk_ is the factor scores of the FA and $W_{ij}$ is the weight matrix.

The absolute scores of factor analysis are calculated by subtracting the factor scores of the zero-sample from the factor scores of real samples:

${AFS}_{jk} = {Fs}_{jk}- {Fs}_{j0}$ (Eq. S8)

Multiple linear regression (MLR) analysis was performed by regressing daily PM_2.5_ concentrations against absolute factor scores:

${PM}_{2.5}= \beta_{0}+ \sum_{i=1}^{m} (\beta_{j}\times{AFS}_{jk})$ (Eq. S9)

Where PM_2.5_ represents the concentrations of fine particulate matter (µg m^-3^), $\beta_{0}$(intercept) represents the PM_2.5_ mass not explained by the factors (unaccounted sources), and $\beta_{j}\times{AFS}_{jk}$ represents the contributions of factor *j* to PM_2.5_ concentrations (µg m^-3^).

The contribution rates of the sources identified by FA were calculated as described by Hou and collaborators (2022):

${PC}_{ij}=\frac{\left| \beta_{j}\times\bar{{AFS}_{jk}} \right|}{\left| \beta_{0} \right|+\sum_{j=1}^{J} \left| \beta_{j}\times\bar{{AFS}_{jk}} \right|}\times100\%$ (Eq. S10)

The percentual contribution from unaccounted sources is described by equation S11:

${PC}_{ij}=\frac{\left| \beta_{0} \right|}{\left| \beta_{0} \right|+\sum_{j=1}^{J} \left| \beta_{j}\times\bar{{AFS}_{jk}} \right|}\times100\%$ (Eq. S11)

**REFERENCES**

Brown S, Minor H, Brien TO, Hameed Y, Feenstra B, Kuebler D, Wetherell W, Day R, Tun R, Landis E, Rice J (2019). Review of Sunset OC / EC Instrument Measurements During the EPA ’ s Sunset Carbon Evaluation Project. Atmosphere, 10:287. https://doi.org/10.3390/atmos10050287.

George SK, Nair PR, Parameswaran K, Jacob S, Abraham A (2008). Seasonal Trends in Chemical Composition of Aerosols at a Tropical Coastal Site of India. J. Geophys. Res. Atmos. 113:1–15. <https://doi.org/10.1029/2007JD009507>.

Hou X, Gao W, Zhang M, Xia R, Chen X, Deng Y (2022). Source apportionment of water pollutants in Poyang Lake Basin in China using absolute principal component score – multiple linear regression model combined with land-use parameters. Front. Environ. Sci.,10:924350. https://doi.org/10.3389/fenvs.2022.924350.

IAG (2022). Institute of Astronomy, Geophysics and Atmospheric Sciences of the University of São Paulo. http://www.estacao.iag.usp.br. Accessed 26 September 2022

Revelle W (2025). Package ‘psych’. Available at: https://personality-project.org/r/psych/psych-manual.pdf accessed 10 March 2026.

Shrivastava A, Gupta VB (2011). Methods for the determination of limit of detection and limit of quantitation of the analytical methods. Chronicles Young Sci., 2:21–25. https://doi.org/10.4103/2229-5186.79345.

Thurston GD, Spengler JD (1985). A quantitative assessment of source contributions to inhalable particulate matter pollution in metropolitan Boston. Atmos. Environ. 19:9–25. https://doi.org/10.1016/0004-6981(85)90132-5.

U.S. EPA (2023). Regional Screening Levels (RSLs) - Generic Tables. Summary Table. <https://www.epa.gov/risk/regional-screening-levels-rsls-generic-tables> Accessed 20 August 2023.
